# Global variation in seed covering structure hardness of woody species with orthodox seeds

**DOI:** 10.1093/aob/mcaf027

**Published:** 2025-02-27

**Authors:** Anne M Visscher, Pablo Gómez Barreiro, Marybel Soto Gomez, Angelino Carta, Udayangani Liu, Yu Wu, Deshika Muthuthanthirige, Félix Forest, Sian McCabe, Hugh W Pritchard

**Affiliations:** Royal Botanic Gardens, Kew, Wakehurst, Ardingly, West Sussex, RH17 6TN, UK; Royal Botanic Gardens, Kew, Wakehurst, Ardingly, West Sussex, RH17 6TN, UK; Royal Botanic Gardens, Kew, Richmond, London, UK; Department of Biology, Botany Unit, University of Pisa, Pisa, Italy; Royal Botanic Gardens, Kew, Wakehurst, Ardingly, West Sussex, RH17 6TN, UK; Royal Botanic Gardens, Kew, Wakehurst, Ardingly, West Sussex, RH17 6TN, UK; College of Forestry, Nanjing Forestry University, 159 Longpan Road, Xuanwu District, Nanjing, Jiangsu 210037, PR China; Royal Botanic Gardens, Kew, Wakehurst, Ardingly, West Sussex, RH17 6TN, UK; Ecology and Conservation Biology, Institute of Plant Sciences, University of Regensburg, Regensburg, Germany; Royal Botanic Gardens, Kew, Richmond, London, UK; Royal Botanic Gardens, Kew, Wakehurst, Ardingly, West Sussex, RH17 6TN, UK; Royal Botanic Gardens, Kew, Wakehurst, Ardingly, West Sussex, RH17 6TN, UK; Chinese Academy of Sciences, Kunming Institute of Botany, Yunnan, China

**Keywords:** Seed dormancy, seed dispersal, seed coat, fruit pericarp, puncture force, seed morphology, precipitation, physical dormancy, hardseeded, desiccation tolerance, woody plants, seed covering structure hardness

## Abstract

**Background and Aims:**

Seed covering structure hardness may play a role in defence/predation, physical dormancy and *in situ* longevity/persistence. However, research to date has been limited regarding quantification methods, plant diversity and geographical distribution. In this study, we determined global variation in seed covering structure hardness of woody species with desiccation-tolerant seeds and analysed its relationships with relevant climatic variables, seed traits and ecological processes.

**Methods:**

We measured seed covering structure hardness of 476 species from 459 genera and 113 families using puncture force. We used phylogenetically informed regressions to test covering structure hardness against potential quantitative predictors [19 climate variables (*n* = 405), ten seed morphological traits (*n* = 413), elevation (*n* = 405), genus age (*n* = 375)] and response variables [*ex situ* seed longevity (*n* = 67), germination rate (*n* = 82), species distribution/range size (*n* = 403)]. Categorical predictors [geographical region (*n* = 444), plant lifeform (*n* = 428), seed dormancy type (*n* = 146), seed physical dormancy in the family Fabaceae (*n* = 76), dispersal unit or mechanism (*n* = 484), fruit type (*n* = 427)] were tested using pairwise comparisons.

**Key Results:**

Seed covering structure hardness ranged from 0.13 to 366.38 N and seed and fruit (seed/fruit) size, seed/fruit roundness, seed/fruit colour (lightness) and precipitation of the driest quarter were significantly associated with hardness. In addition, dormancy types (vs non-dormancy), dispersal as fruit (vs seed) or certain fruit types (fleshy vs dry, drupes vs other types), as well as animal dispersal (vs other mechanisms) showed higher levels of hardness. Furthermore, covering structure roundness was higher in animal-dispersed seeds/fruits (vs other dispersal strategies). Finally, covering structure hardness was shown to predict germination rate but not *ex situ* seed longevity or species range size.

**Conclusions:**

Our results suggest roles for morphology, dormancy, dispersal and precipitation in explaining part of the global variation in seed covering structure hardness of woody species with orthodox seeds. However, we showed that the presence of physical dormancy does not always imply having a harder covering structure than non-dormant seeds and therefore terms such as ‘hardseeded’ or ‘hard coat’ should no longer be used as synonyms for this trait.

## INTRODUCTION

### Seed covering structures and their functions

Seeds are formed during the reproductive life stage of plants and consist of an embryo, nutritive tissue and one or more covering structures. Seed covering structures may have multiple functions, including defence, dispersal and germination regulation (e.g. dormancy) (Finch-Savage and Leubner-Metzger, 2006; Chen and Moles, 2018). However, little is known about the global variation in seed covering structure hardness and the potential reasons underlying natural diversity in this trait.

In angiosperms, both seeds and fruits can be the unit of dispersal, whilst gymnosperms lack fruit structures (Linkies *et al.*, 2010). As part of the dispersal unit, seeds can therefore be covered by a seed coat only, or by a seed coat plus fruit layers (Carta *et al.*, 2024). Seed coats typically develop from one or two (rarely three) integuments (layers surrounding the nucellus) whilst pericarps (fruit coats) develop from the mature ovary wall and other flower tissues (Linkies *et al.*, 2010; Coen and Magnani, 2018; Rudall, 2021).

Hard encapsulations may protect seeds at some or all stages during their life span, such as development, dispersal and post-dispersal prior to germination (Huss and Gierlinger, 2021). Cracked or weak seed coats permit nutrient and electrolyte leakage, as well as rapid water uptake, which can encourage the growth of microorganisms and lead to imbibitional injury respectively (Mohamed-Yasseen *et al.*, 1994). Mutants of *Arabidopsis thaliana* seeds defective in genes regulating seed coat composition and properties exhibited decreased longevity and increased permeability under laboratory conditions, suggesting that the primary role of the seed coat is to isolate the embryo from environmental factors such as oxygen and water (Zinsmeister *et al.*, 2020). In addition to having a functional role in defence, the seed coat also acts as a channel for transmitting environmental cues to the interior of the seed (Radchuk and Borisjuk, 2014). Seed covering structures are furthermore known to impose physiological, physical or mechanical dormancy (Finch-Savage and Leubner-Metzger, 2006; Neya *et al.*, 2008; Sperber *et al.*, 2017). For example, seed physical dormancy (PY) caused by a water-impermeable seed (or fruit) coat is known to occur in at least 15 families of angiosperms (Baskin *et al.*, 2000).

### Quantification of seed covering structure hardness

It is known subjectively, through physical handling of seeds and fruits, that considerable variation exists in covering structure hardness between species. However, few studies have quantified this trait and quantification methods have been limited. For example, Blate *et al.* (1998) tested seed coat hardness of 40 tree species using a fingernail and knife and divided this trait into three broad categories (soft, hard, very hard). Wiggers *et al.* (2017) used scarification time as an estimate of seed coat hardness for six legume species. Sinha and Voisey (1978) measured seed coat puncture resistance for 42 cultivars of common crops to approximate seed penetration by weevils that can infest stored grain. However, whole seeds were used for the tests rather than separated covering structures (Sinha and Voisey, 1978). Whole seeds were also used by Zalamea *et al.* (2018) in a study of 16 neotropical lowland pioneer tree species for measuring seed coat rupture with a compression probe. When whole seeds are measured, covering structure hardness is affected by two components, namely the hardness of the seed coat and the hardness of the seed interior (Frączek *et al.*, 2005). For the current study, we aimed to quantify the global variation in covering structure hardness of woody species through puncture force measurements of separated coats or fruit layers.

### Potential explanations for variation in seed covering structure hardness: research objectives and hypotheses

Although seed covering structure hardness has so far not been measured at scale using direct quantification of separated covering structures, several climatic variables, seed traits as well as ecological processes have been found to be (in)directly or subjectively associated with hardness. These associations highlight several variables that should be considered in this first global study aiming to understand natural diversity in seed covering structure hardness within a phylogenetic framework. Below we introduce these variables and the hypotheses tested in this study.

### Quantification of variation in seed covering structure hardness

Given the limitations of quantification methods used in previous studies (see above) and the suggestion that covering structure hardness is affected by the hardness of the seed interior when whole seeds are measured (Frączek *et al.*, 2005), our first objective was to compare measurements of covering structures using whole seeds versus separated structures. Our hypothesis was that hardness values ascribed to the covering structure layers using whole seeds are significantly different from values registered when measuring the layers separately.

### Climate (temperature and precipitation), geographical zones and plant lifeform

Climate, and in particular precipitation, has been shown to play a role in explaining the key evolutionary process of natural selection (Siepielski *et al.*, 2017). Mature seed traits that have previously been linked to variation in climate on a global scale include seed mass (Moles *et al.*, 2005) and seed desiccation tolerance (Tweddle *et al.*, 2003). For example, in tropical and subtropical zones, the proportion of species with desiccation-sensitive seeds declines as the habitat becomes drier, (i.e. from 46.6 % in evergreen rainforest to 2.2 % in hot desert and semi-desert; Tweddle *et al.*, 2003). In 16 species that produce desiccation-sensitive (recalcitrant) seeds from a seasonal tropical forest in Australia, the ratio of seed coat mass to seed mass (relative thickness of covering structures) was found to be negatively correlated with mean monthly rainfall at mean time of seed dispersal, potentially allowing more rapid germination in the presence of precipitation (Hill *et al.*, 2012; Obroucheva *et al.*, 2016). However, our study focuses on woody species with desiccation-tolerant rather than desiccation-sensitive seeds. In addition, it is unclear what the effects of precipitation are on absolute covering structure thickness (and therefore potentially absolute hardness), although arid environments seem to favour the development of thicker coats in a species of Fabaceae (Richard *et al.*, 2018). Despite limited existing knowledge, we hypothesized that seed covering structure hardness is negatively associated with precipitation. Furthermore, we predicted that there would be no differences in covering structure hardness between tropical and temperate zones (in line with findings for relative covering structure thickness; Chen and Moles, 2018) or plant lifeforms (due to most of our species being woody). However, geographical zones and plant lifeform are known to be correlated with variation in other plant and seed traits (Moles *et al.*, 2005; Visscher *et al.*, 2022) and were therefore also tested in this study.

#### Seed/fruit morphology and colour.

From a physical point of view and in absolute terms, larger seeds appear to have a higher potential for thicker – and therefore potentially harder – covering structures than smaller seeds. Indeed, Wada and Reed (2011) showed that seed coat thickness is positively correlated with both seed coat hardness and seed weight (an indicator of size) in *Rubus* species. Seed covering structure hardness may be partly due to geometry and play a role in predation and animal dispersal as spherical shape was found to increase the fracture resistance of different animal-dispersed plant shells due to geometric stiffening (Huss *et al.*, 2020). There are also indications that seed coats with darker colours may be more mechanically resistant than those with lighter colours, at least within the same species (Prasad and Weigle, 1976; Mohamed-Yasseen *et al.*, 1994). Darker seed coats were shown to have higher phenol and flavonoid contents in *Phaseolus vulgaris*, but whether these phytochemicals are the reason for the reduced susceptibility to cracking of black-coated cultivars was not examined (Prasad and Weigle, 1976). Based on the above, our hypotheses were therefore that seeds with harder covering structures are (1) larger, (2) more spherical (rounder) and (3) darker in colour.

#### Seed dormancy.

‘Hardseededness’ or ‘hard coat’ are regularly used terms in the scientific literature to refer to physical seed dormancy (Paulsen *et al.*, 2013; Chamorro and Moreno, 2019). However, to our knowledge, it is currently unclear whether water-impermeable covering structures are always harder than water-permeable structures. Our objective was to compare seed covering structure hardness between species with impermeable and permeable structures to test our hypothesis that physically dormant (impermeable) species have harder covering structures than non-dormant (permeable) species.

Whilst physical dormancy and ‘hardseededness’ or ‘hard coat’ appear to be synonymous, there do not seem to be any indications that this is the case for all seed dormancy types. Therefore, our second dormancy-related hypothesis was that seeds with PY have harder covering structures than non-dormant seeds whereas other dormancy types do not show differences in covering structure hardness compared to each other or non-dormant seeds.

#### Seed longevity.

Coat thickness and hardness may affect seed longevity/persistence. For example, a study of 13 European weed species showed that seed mortality in the soil decreased with increasing seed coat thickness (Gardarin *et al.*, 2010). In addition, coat hardness was strongly correlated with longevity of experimentally buried seeds of 28 *Vaccinium* species (Hill and Vander Kloet, 2005). Whether covering structure hardness also correlates with *ex situ* longevity is not clear, although increased seed coat permeability is typically associated with reduced seed longevity/enhanced ageing under laboratory conditions (Renard *et al.*, 2020; Zinsmeister *et al.*, 2020), and seeds with water-impermeable covering structures are often referred to as ‘hard’. Our hypothesis was that seeds with harder covering structures show slower germination decline (enhanced longevity) during *ex situ* seed storage.

#### Seed germination rate.

It is not clear whether covering structure hardness affects germination rate. For example, *Lepidium didymum* seeds removed from fruit structures (which impose mechanical dormancy) germinated more rapidly than those left in the fruits (Sperber *et al.*, 2017), but without the availability of larger data sets it is unclear whether the reported structure hardness is relatively high or not. However, if physically dormant seeds are indeed shown to have harder covering structures than non-dormant seeds, this may indirectly lead to slower germination rates. Our hypothesis therefore was that seeds with harder covering structures have lower germination rates.

#### Seed dispersal and species distribution/range size.

There are indications that fruit layers covering seeds can be particularly hard. For example, fruits with a hardened endocarp are called drupes and the large, lignified endocarp that surrounds the seed is commonly called the stone (Dardick and Callahan, 2014). There is also evidence that seeds/fruits dispersed by animals are rounder and that this leads to increased fracture resistance as well as increased gut survival (Huss *et al.*, 2020; Lovas-Kiss *et al.*, 2020). It is less clear whether coat hardness affects the rate of predation. For example, Blate *et al.* (1998) found that predation rates of seeds from 40 rainforest tree species in Indonesia were negatively associated with the thickness and hardness of the seed coat. However, Gong *et al.* (2015) surveyed the predation of 48 600 seeds from 30 species for three consecutive years in a natural pine forest and demonstrated that seed coat hardness did not significantly affect seed predation by rodents. Based on the above, we hypothesized that harder covering structures are found in (1) seeds covered in fruit layers, (2) seeds as part of drupes covered with hardened endocarp and (3) seeds/fruits dispersed by animals. We also hypothesized that seed covering structure hardness is negatively associated with species distribution range. For example, when studying 234 tree species, Morin and Chuing (2006) found that species tend to have larger ranges when they are closer to the poles, are successionally seral, have small and light seeds, and have short generation times. We therefore argued that seeds with less hard covering structures have the potential to be lighter and therefore to be dispersed further (e.g. by wind).

#### Genus age.

Seed coat thickness is partly determined by integument number and angiosperms are bitegmic in the ancestral state, with unitegmic ovules having evolved several times independently and found to be present in 22 % of angiosperm families analysed (Lora *et al.*, 2015; Coen and Magnani, 2018; Rudall, 2021). For example, older families in the order Santalales show a higher incidence of bitegmy than younger families (Brown *et al.*, 2010). Seed coat thickness and seed (coat) hardness may be linked. For example, in an analysis of 40 rainforest tree species in Indonesia, Blate *et al.* (1998) observed that seed coat thickness was greatest in seeds with very hard coats, intermediate in seeds with hard coats and lowest in seeds with soft coats. Furthermore, a study of 17 *Rubus* species showed a significant positive correlation between seed coat thickness and seed coat hardness (Wada and Reed, 2011). Our hypothesis was that bitegmy occurs more often in older genera and therefore covering structures from older genera have the potential to be(come) thicker and consequently harder.

In this first global assessment, we measured seed covering structure hardness of 476 woody species from 459 genera and 113 families using puncture force. We then used phylogenetically informed regressions and categorical comparisons to test covering structure hardness against relevant climatic variables, seed traits and ecological processes.

## MATERIALS AND METHODS

### Seed collections and nomenclature

**Fig. 1.**
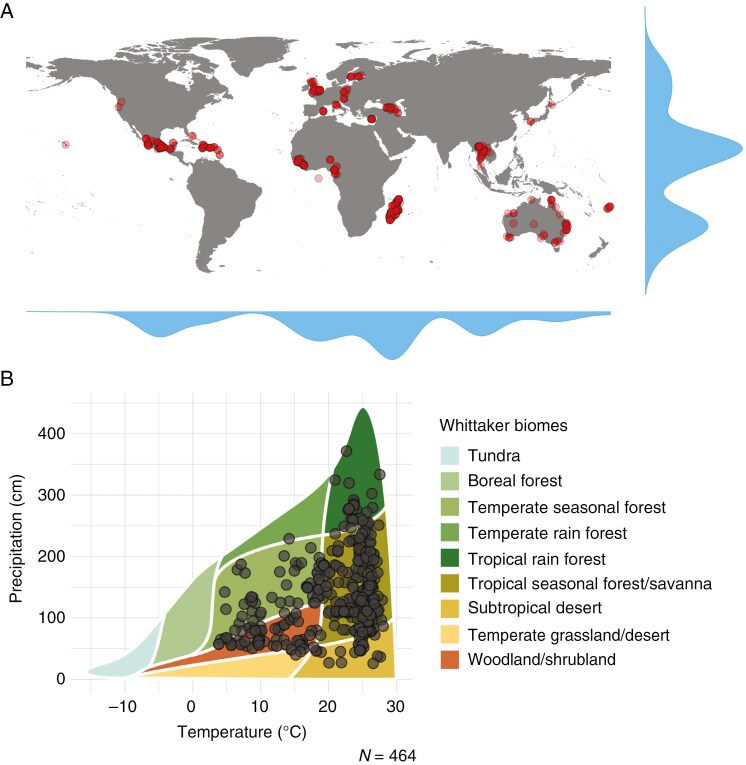
(A) The global distribution of the 484 seed collections (476 species from 459 genera and 113 families) for which covering structure hardness was measured in this study using puncture force. The latitudinal and longitudinal distribution density of the seed collections is shown in blue. (B) The distribution across biomes (as defined by the Whittaker classification scheme) of 464 of the 484 seed collections measured for covering structure hardness in this study.

We focused on predominantly woody species with orthodox (desiccation-tolerant) seeds held in the Millennium Seed Bank (MSB) of the Royal Botanic Gardens, Kew. The seed collections are part of the Global Tree Seed Bank Programme (GTSBP: 715 collections) and the UK National Tree Seed Project (UKNTSP: 32 collections) of the MSB Partnership. To cover a wide range of taxonomic diversity, we selected one species per genus (based on seed number and germination data availability) of GTSBP and UKNTSP collections held in the MSB, giving a total of 747 seed collections representing 737 species, 707 genera and 159 families (Supplementary Data file 1). Several species and genus duplicates exist due to both the addition of seed collections from higher latitudes that were less represented (UKNTSP) and taxonomic name changes that occurred during the study. Seed collections received by the MSB are usually associated with taxonomic identities from family to species level and beyond, and these are regarded as the original taxonomic names. These names are checked against the World Checklist of Vascular Plants made available in Plants of the World Online (POWO, 2022). The certainty of taxonomic name identification for each collection is given as either ‘verified’ with high degree of confidence or ‘unverified’ with low degree of confidence. For collections with unverified status, where possible, the herbarium specimens and/or images are used to verify their identities. Prior to data analysis we re-extracted the taxonomic names attached to collections to capture any updates that had been made during the study. Any remaining collections with unverified status were treated equally to those with verified status in subsequent analyses. The seed material was donated for safety duplication by ~54 organizations (see Acknowledgments) from 29 countries around the world (Fig. 1A) covering a range of biomes (Fig. 1B).

### Hardness measurements of the seed covering structures

All seed/fruit collections were stored in a dry room (15 % relative and 18 °C) until measurement. The covering structures measured include seed coats as well as fruit layers (the pericarp or parts of it). Preliminary tests of covering structure hardness were performed on both whole seeds/fruits and separated covering structures of 157 collections to assess whether whole seeds/fruits could be used to quantify covering structure hardness or whether measurements needed to be done on separated covering structures. Overall, the hardness of separated covering structures was determined for 484 collections representing 476 species from 459 genera and 113 families (Table 1; Supplementary Data file 1). The ‘*ex situ* storage units’ that we handled may be somewhat different from the dispersal/germination units as seeds or fleshy layers may have been removed from fruits before seed bank storage. Measurements were taken for three to five replicate seeds (two in certain instances) per collection, with one replicate being the covering structure of one seed. A part of each seed covering structure was removed from other seed tissues (e.g. endosperm, embryo) and placed in a convex manner on/in a layer/mould of white tack (UHU, Germany) that was positioned on a sheet of paper held down by magnets. The force required to puncture or break each piece of covering structure was measured using an Instron 5942 machine loaded with a 50-kN cell (Instron, UK). The probes consisted of stainless-steel needles with a tip diameter of 142–368 μm attached to a pin vice. Puncture or breaking force was determined by selecting the maximum applied force (N) for each sample via Bluehill Universal Software (Instron), with the probe seed set to progress at 1 mm min^−1^ and the applied force recorded every 0.01 s following first contact with the sample.

**Table 1.** Data analysis sample sizes: the number of seed collections* with data available for analysis. Column 2 shows the number of seed collections with data available for single data categories described in column 1. Column 4 shows the number of seed collections with data available for multiple data categories described in column 3. Numbers in parentheses in column 4 indicate the number of species with data available for multiple categories that are also represented in the phylogenetic trees used for phylogenetically informed regressions.

Data category (single)	Sample size	Data category (combinations)	Sample size
Seed covering structure hardness (SCSH)	484	SCSH (and phylogenic tree representation: PTR)	484 (422)
Climate variables	713	Climate variables and SCSH (and PTR)	463 (405)
Elevation data	713	Elevation data and SCSH (and PTR)	463 (405)
Location data (for geographical regions)	731	Location data and SCSH	444
Lifeform	649	Lifeform and SCSH	428
Morphological variables	722	Morphological variables and SCSH (and PTR)	474 (413)
		Climate, morphology and SCSH (and PTR)	453 (392)
Dormancy type^†^	214	Dormancy type and SCSH	146
Fabaceae coat permeability (imbibition)	59	Fabaceae coat permeability (imbibition) and SCSH	48
Germination rate	166	Germination rate and SCSH (and PTR)	95 (82)
Germination decline during *ex situ* storage^‡^	117	Germination decline during *ex situ* storage and SCSH (and PTR)	72 (67)
Dispersal unit (seed/fruit)	747	Dispersal unit (seed/fruit) and SCSH	484
Fruit: dry or fleshy	694	Fruit dry or fleshy and SCSH	453
Fruit type	686	Fruit type and SCSH	427
Dispersal mechanism	747	Dispersal mechanism and SCSH	484
		Seed roundness and dispersal mechanism	722
Species distribution (range size)	426	Species distribution and SCSH (and PTR)	426 (403)
Genus age	428	Genus age and SCSH (and PTR)	428 (375)

^*^One collection was measured twice (red and black parts of seed coat): this collection is counted as one collection but has two measurements.

^†^Number of collections with single dormancy categories.

^‡^Number of species.

### Seed/fruit morphological measurements

Seeds from 722 collections (Table 1; Supplementary Data file 1) were imaged using a DSLR camera (Nikon D750 + Nikkor 105 mm) and a backlit copy stand (eVision executive HF, Kaiser Fototechnik) with fluorescent top lights, or a camera (Axiocam 208 colour, Zeiss) mounted on a stereoscope (Stemi V11, Zeiss), depending on seed/fruit size. External morphological traits [area, perimeter, maximum and minimum feret (the distance between any two points along the selection boundary), circularity, aspect ratio, roundness, and colour channels *a*, *b* and *L* associated with CIELAB colour space] were then obtained with Fiji/ImageJ v.1.54f (Schindelin *et al.*, 2012).

### Seed imbibition measurements

Seed imbibition measurements were done for species in the family Fabaceae to test whether impermeable seed coats are generally harder than permeable seed coats. We selected the family Fabaceae as this is the largest plant family known to have species with PY and the family most represented at genus and species levels in our study (Supplementary Data files 1 and 6). Another reason for selecting Fabaceae species was that they could be compared on the basis of seed coats only, rather than a mixture of different types of covering structures. To increase the data set available for analysis based on the literature (Baskin and Baskin, 2014), seeds from 59 Fabaceae species with no known dormancy information (Table 1; Supplementary Data file 2) were subjected to water imbibition tests to determine whether they were predominantly impermeable (physically dormant) or (more) permeable (non-physically dormant). The imbibition tests were performed as follows: three replicate samples of ten seeds each were submersed in water. Replicates were weighed before (dry) and after submersion in water (at 24 h, 48 h and 7 d, followed by 7 d or sometimes longer intervals when collections appeared to be potentially impermeable). Submersed seeds were surface-dried before weighing. For potentially impermeable collections for which weighing continued beyond the first 7 d, germinated and enlarged seeds were counted and removed before weighing.

In species with PY, fractions of seeds can be dispersed with water-permeable seed coats (Meisert, 2002; Zalamea *et al.*, 2015). For this study, collections/species were considered physically dormant when fewer than 20 % of seeds had germinated and/or become enlarged and water uptake by seed weight for the remaining seeds was ≤5 % over 79–121 d. This approach is in line with previous studies (e.g. Chandra *et al.*, 2020).

### Collection of existing environmental, plant and seed/fruit trait data

#### Climate, elevation and location data collection.

Location data were extracted from MSB’s Seed Bank Database (now EarthCape Integrated Collection Management System: ICMS). These coordinates were used to obtain bioclimatic variables (www.worldclim.org/data/bioclim.html) and elevation data with a 30′ resolution via WorldClim v.2 (Fick and Hijmans, 2017) for 713 seed collections (Table 1; Supplementary Data file 1).

#### Plant lifeform.

Plant lifeforms are listed in Plants of the World Online (POWO, 2022) following the classification of Raunkiær (1934). We extracted lifeform data for 649 seed collections by matching their Latin names with POWO (Table 1; Supplementary Data file 1). For data analysis, lifeform categories were grouped into higher-level woody and non-woody categories.

#### Fruit type, seed dispersal form and dispersal strategies.

We conducted a thorough investigation into fruit type and seed dispersal form and strategies, utilizing a multidisciplinary approach to compile data on all 747 seed collections from scientific publications, Royal Botanic Gardens, Kew databases, online botanical repositories, and national floras (Table 1; Supplementary Data files 1 and 3). Our objective was to identify the fruit type and primary dispersal unit of each species and their mode of dispersal. To achieve this, we systematically classified different fruit types into simple, aggregate or composite categories. Subsequently, we examined information on the external morphology of seeds or fruits, recognizing their importance in influencing various modes of dispersal and considering critical factors such as (in)dehiscence of the fruit. Additionally, we determined the dispersal unit of a species by evaluating the mode of dispersal and/or the agents involved, particularly in cases where direct information on diaspores was unavailable. In instances where species-level data were unavailable, our analysis extended to encompass the genus or family level to ensure comprehensive coverage of seed dispersal strategies. Further, we considered whether seeds are released from a decaying ripe fruit, with the fruit itself serving as the diaspore. The determination of whether a species is dispersed as a seed only or as a seed in fruit tissues is visualized in Fig. S1 (Supplementary Data file 4). Note that the fruit type and seed dispersal form do not necessarily always correspond to the *ex situ* storage form in which the covering structure hardness was measured [e.g. seeds of some collections may have been expelled (through natural processes) or artificially removed (for *ex situ* storage purposes) from the fruit tissues they developed and/or were dispersed in].

The dispersal strategies were classified into five distinct groups: autochory, zoochory, anemochory, hydrochory and myrmecochory. When direct information on dispersal categories was not available, strategies were determined by examining seed and fruit characteristics, although we realize that this approach is not as accurate as using field observations of dispersal movements (Green *et al.*, 2022). For instance, we considered wings or papery structures as indicating anemochory, while we considered fruits that naturally dehisce as a trait commonly associated with autochory. Furthermore, we considered distinctive traits facilitating the dispersal of dehiscent fruits, such as seeds enclosed in red or orange arils, often indicative of zoochory. Fleshy fruit parts were identified as indicators of zoochory. In cases where certain species demonstrated multiple dispersal mechanisms, priority was given to the category with the most available data. If information was accessible for both dispersal mechanisms, both categories were incorporated into the analysis. For data analysis, only categories with more than ten occurrences were included, which resulted in myrmecochory being merged with zoochory and no species with multiple (potential) dispersal mechanisms being represented.

#### Seed dormancy.

Seed dormancy type data were retrieved from the reference work by Baskin and Baskin (2014) for 240 seed collections. Data analysis was limited to species with single dormancy categories (*n* = 214) to avoid potentially uncertain or contradictory results (e.g. species listed as both non-dormant and with a type of dormancy) (Table 1; Supplementary Data file 1). The single dormancy categories included in the analysis were non-dormant (ND), physically dormant (PY), physiologically dormant (PD) or morphologically dormant (MD).

#### Germination decline during ex situ storage.

To review the longevity of accessions during storage, we examined the change in relative germination (delta-RG or dRG) over time. The RG for each test is estimated as a percentage of seeds that are germinated with healthy radicles out of true seeds sown with embryos. The number of true seeds sown is calculated by subtracting the ungerminated empty and infested seeds from the total number of seeds sown. For a successful test, the expected overall germinability must be ≥85 %. Usually, the first round of germination tests (initial tests) is carried out once the accessions are stored at −20 °C (post-storage tests) for at least 7 d, preferably within 3 months of banking. Non-routine pre-storage tests are carried out when accessions are suspected to be short-lived seeds. If the first round of tests is successful and the accession contains enough seeds, the longevity of accessions is monitored during their life cycle in storage through germination retests, at least every 5-, 10- or 20-year period (1st, 2nd, 3rd retests, etc.), depending on expected longevity. The ICMS database (RGB Kew) captured in-depth data on seed germination tests, and these were extracted in March 2022. For the studied plants that are listed to species level, we extracted data for any accession that has a matching species name. For those listed only up to genus level (i.e. genera not represented in the previous matching), we extracted data for any accession with a matching genus. The data set was further narrowed down by eliminating germination tests that were not relevant to −20 °C storage, experimental tests and tests with less than ten true seeds sown and by identifying accessions with at least one completed post-storage test. To review the germinability of accessions after storage, we examined the most recent round of post-storage tests which reflects the current physiological status and then selected the highest RG and assigned it to a size class (0, 1–10, 11–20, etc.). Depending on how long these accessions are stored at −20 °C, the most recent round could be an initial, earlier or later retest. There were 408 accessions representing 117 species (Table 1) with more than one round of tests and with a germination test ‘pass’ (≥85 % final germination) result at the earlier test. The highest RG achieved for these accessions at the earlier round of tests (pre- or post-storage test) was compared against that of the most recent round of tests, and the calculated differences in RG (Supplementary Data file 1) were used for further analyses. RG values were averaged when several values were available for the same species.

#### Germination rate.

Germination rate data were collected from the ICMS database (RGB Kew), which calculates germination rate (the index of germination rate/speed) according to Liu *et al.* (2020). We selected germination rate data for germination tests with a ‘pass’ (≥85 % final germination) result only. When there were multiple germination tests with a ‘pass’ result for a particular collection, we selected the test with the fastest germination rate to capture the most optimal germination conditions tested. Germination tests may have included treatment steps, such as seed dormancy breaking treatments. Germination rate data were available for 166 seed collections (Table 1; Supplementary Data file 1).

#### Species distribution (range size) and genus age.

Species distribution range data (km^2^) were obtained from Carta *et al.* (2022). Range size data were available for 426 seed collections with seed covering hardness data (Table 1; Supplementary Data file 1). We used genus ages provided in Carta and Escudero (2023) who considered the stem age because the crown is not always present (i.e. in monospecific or poorly sampled genera). Genus age data were available for 428 seed collections with seed covering hardness data (Table 1; Supplementary Data file 1).

### Data analyses

Linear regressions were used to study the individual relationships between log_*e*_ *x* (hereinafter referred to as *log*)-transformed response variable ‘seed covering structure hardness’ (‘SCSH’) and *log*-transformed independent variables (morphological traits, climatic and elevation data, and genus age). To account for phylogenetic relationships in correlation analyses, these models were repeated using the function *phylolm* (λ model), provided by the *phylolm* R package (Ho and Ané, 2014). Due to most of the species not being represented in large-scale phylogenetic trees (e.g. Smith and Brown, 2018), taxonomic uncertainty was taken into account by performing each individual model against 100 phylogenetic trees in which missing species were randomly imputed to their respective genus (Forest, 2023). Each species was represented once (first instance) in the *phylolm* analyses.

*Log*-transformed species distribution range, change in relative germination (dRG) and germination rate were studied as dependent variables against ‘SCSH’ following the methods above. Variables with values ≤0 were transformed into positive values by either adding the minimum value necessary to transform all variables to positive numbers, or, when all values where negative (e.g. dRG), by using the absolute number.

The combined effect of multiple *log*-transformed morphological traits, climate and elevation on *log*-transformed ‘SCSH’, including phylogenetic relatedness information (applied following the same method used for individual traits), was studied using linear relationships for non-colinear variables (cutoff = 0.6). The correlations between variables are shown in Supplementary Data Fig. S2. The best minimal model (without considering interactions between variables due to computational constraints) for each of the available 100 phylogenetic trees was chosen based on the lowest Akaike information criterion (AIC) value and the lowest number of degrees of freedom for models within delta 2 from the one with the lowest AIC.

Differences in ‘SCSH’ for categorical variables were studied using pairwise comparison. A Shapiro–Wilk test determined that *log*-transformed ‘SCSH’ for each of the categorical variables (except for dormancy in the family Fabaceae) was non-parametrically distributed. Thus, differences between groups were studied using a Wilcoxon rank-sum/Mann–Whitney *U* test or *t*-test (when only two groups were present) and Kruskal–Wallis followed by Wilcoxon rank-sum/Mann–Whitney *U* test. Groups with ten or fewer samples were not included in the analysis.

Correction for multiple testing was done for omnibus hypotheses [hypotheses including several individual (surrogate) nulls] as recommended by García-Pérez (2023). Omnibus hypotheses in this study include some of the categorical variables (with more than two groups), and the combined regression model. We used the Holm method to correct for multiple testing and we have indicated in the Results and figure legends if this correction changed the significance outcome. As this is a first, and therefore exploratory, global study, we set the alpha level at 0.05 to avoid increasing the Type-II error rates.

The number of collections for which trait data were available for the analyses described in this section are listed in Table 1. The analyses were done in R 4.4.1 (R Core Team, 2024). A comprehensive list of R packages used in this study is available within files provided in a replication package (see Data availability).

## RESULTS

### Variation in seed covering structure hardness

**Fig. 2.**
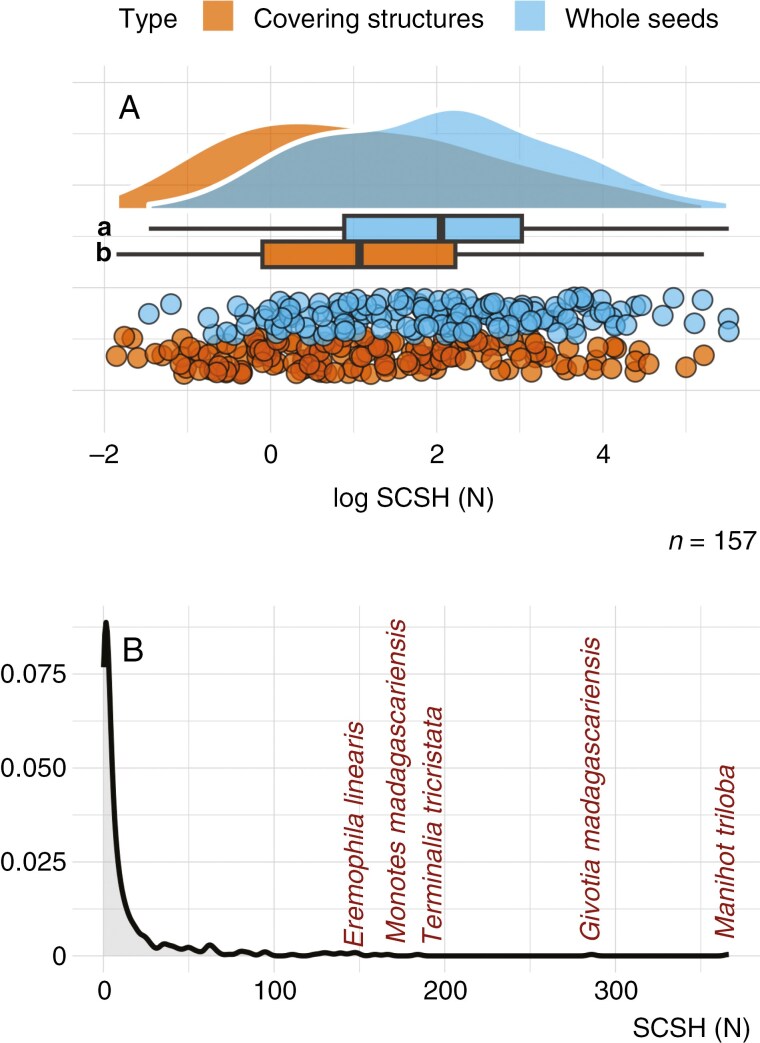
(A) Plots showing puncture force measurements [seed covering structure hardness (SCSH)] for 157 seed collections of whole seed versus covering structure only (both measurements were done on the same collections). Differences between groups were tested using a Wilcoxon rank-sum/Mann–Whitney *U* test. (B) Plot showing the distribution of all seed covering structure hardness data (*n* = 484 seed collections). The five species with the hardest measurable seed covering structures are indicated.

Preliminary tests of 157 collections indicated that the use of whole seeds rather than separated covering structures led to significantly higher (1.7×) estimates of covering structure hardness on average (Fig. 2A). This effect was not equally distributed across all seed collections, with separated covering structures being less hard, similar or harder compared to the estimates based on whole seeds (Supplementary Data Fig. S3). One of the reasons for this unequal effect may be variation among species in hardness of the interior seed tissues, which we observed but did not quantify in this study. An additional possible reason is errors in selecting the peak (potentially due to the presence of many peaks or a peak not having been registered separately from other tissues) in the puncture force graphs that corresponds to the (entire) covering structure when using whole seeds. Because the aim of our study was to analyse variation in seed covering structure hardness across species, we measured seed covering structures separately from the rest of the seed tissues to rule out the effects of variation in interior seed tissue hardness and puncture force estimation errors. Of the 685 collections assessed, covering structure hardness could not be determined for 201 collections due to the seeds being too small (most measured seeds were >1.15 mm^2^), or because the covering structures were too hard (>400 N) or could not be removed from the endosperm and embryo tissues (Supplementary Data files 1 and 5). Covering structure hardness ranged from 0.13 to 366.38 N across the 484 seed collections measured (Fig. 2B), with 16.1 ± 35.1 N being the average (±s.d.) and 3.63 N the median. Figure S4 shows covering structure hardness averaged per represented family across a family-level phylogenetic tree with the number of species sampled per family indicated (Supplementary Data file 6).

### Relationship between seed covering structure hardness and physical dormancy in the family Fabaceae

The large number of Fabaceae genera/species represented in our study allowed us to perform a within-family case study on seed coat hardness and its relationship with seed coat (im)permeability and therefore to quantitatively assess whether PY (impermeability) is synonymous with hardness as commonly assumed in the literature. There were 48 seed collections identified as Fabaceae that were measured for covering structure hardness but that did not have dormancy information available according to Baskin and Baskin (2014). Imbibition tests for these 48 seed collections showed that eight of the collections are predominantly characterized by a water-impermeable seed coat (>80 % of seeds) and therefore have physical dormancy, whilst the remaining 40 collections (hereafter called ‘non-dormant’) have either lower levels of impermeability or fully water-permeable coats.

**Fig. 3.**
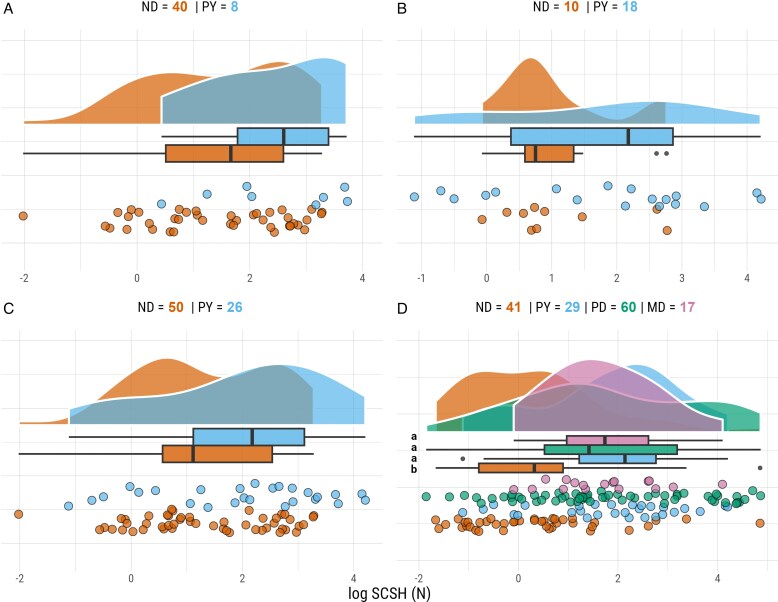
Graphs showing dormancy in relation to seed covering structure hardness (SCSH) (*log*): (A) ND versus PY in the family Fabaceae based on seed coat permeability (imbibition) tests; (B) ND versus PY in the family Fabaceae based on Baskin and Baskin (2014); (C) ND versus PY in the family Fabaceae based on both imbibition tests and Baskin and Baskin (2014); and (D) all seed dormancy categories across 146 collections based on Baskin and Baskin (2014). Differences between groups were analysed using a *t*-test (A–C) or Kruskal-Wallis followed by Wilcoxon rank-sum/Mann-Whitney *U* test and Holm correction (D).

In addition, there were 28 Fabaceae collections that were listed as either non-dormant or physically dormant by Baskin and Baskin (2014). Figure 3A and B show the ranges in average covering structure hardness for non-dormant and physically dormant seed collections for the data set based on imbibition tests as well as for the data set derived from Baskin and Baskin (2014). In addition, Fig. 3C shows the ranges for the total set of 76 seed collections. The *t*-tests showed that, although PY groups have higher averages, the hardness of the species groups with predominantly impermeable seed coats is not significantly different from that of the groups with permeable coats (Fig. 3A–C).

### Seed covering structure hardness and its relationships with climate and plant/seed traits

**Fig. 4.**
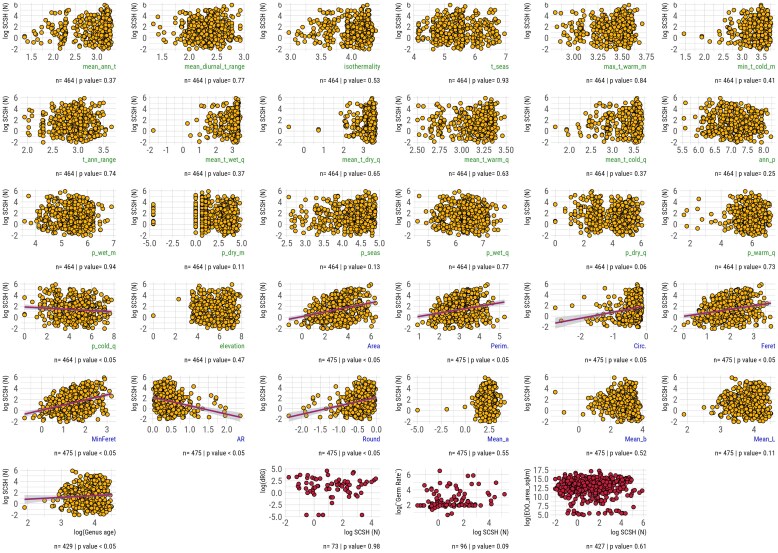
Overview of the seed covering structure hardness (SCSH) data (*log*) plotted against all individual continuous traits (*log*). Panels show results from ordinary linear regression analyses with significant results indicated with regression lines. Graphs show climate (19) and elevation variables (indicated with green *x*-axis titles) followed by morphological traits (ten: indicated with blue *x*-axis titles) and subsequently other plant and seed traits. See the legend to Fig. 5 for the full names of the continuous traits. Graphs with variables considered potential predictors of SCSH are indicated in yellow and graphs with SCSH considered a potential predictor of other plant/seed traits are indicated in red.

**Fig. 5.**
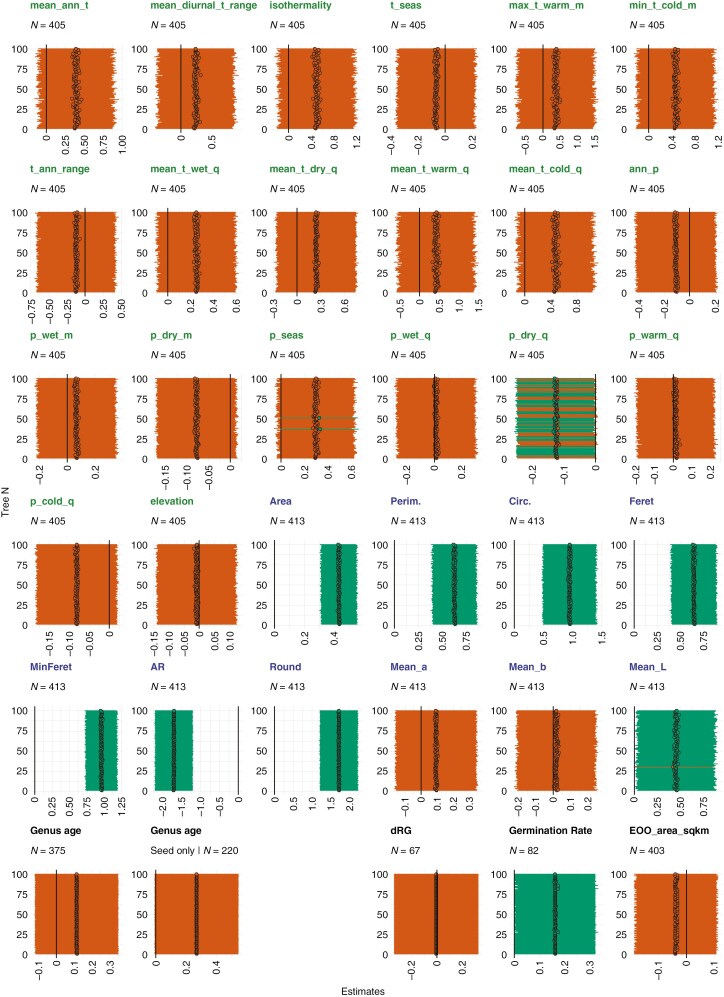
Overview of phylogenetically informed regression analysis results (using *phylolm*) for seed covering structure hardness (SCSH) (*log*) versus each continuous variable (*log*). The individual panels show the *phylolm* estimates and confidence intervals for 100 phylogenetic trees by Forest (2023) (each horizontal line and dot represents one individual tree). Significant (*P* < 0.05) results are indicated in green and non-significant results are indicated in orange. Seed covering structure hardness was tested as the response variable for mean_ann_t (annual mean temperature), mean_diurnal_t_range (mean diurnal range), isothermality, t_seas (temperature seasonality), max_t_warm_m (max temperature of warmest month), min_t_cold_m (min temperature of coldest month), t_ann_range (temperature annual range), mean_t_wet_q (mean temperature of wettest quarter), mean_t_dry_q (mean temperature of driest quarter), mean_t_warm_q (mean temperature of warmest quarter), mean_t_cold_q (mean temperature of coldest quarter), ann_p (annual precipitation), p_wet_m (precipitation of wettest month), p_dry_m (precipitation of driest month), p_seas (precipitation seasonality), p_wet_q (precipitation of wettest quarter), p_dry_q (precipitation of driest quarter), p_warm_q (precipitation of warmest quarter), p_cold_q (precipitation of coldest quarter), elevation, area, perim. (perimeter), circ. (circularity), feret (maximum feret), minferet (minimum feret), AR (aspect ratio), round (roundness), mean_a [mean of colour channel *a* (green – red)], mean_b (mean of colour channel *b* (blue – yellow)], mean_l (mean of colour channel *L* (black – white)], genus age and genus age ‘seed only’ (genus age limited to angiosperms dispersed as seeds). In addition, seed covering structure hardness was tested as the predictor for dRG (difference in relative germination during *ex situ* storage), germination rate (in days), and EOO_area_sqkm (extent of occurrence in square kilometres). The 19 climate (and elevation) variables are indicated with green titles and the ten morphological traits with blue titles.

Figure 4 shows the seed covering structure hardness data (*log*) against all individual continuous variables (*log*). Ordinary linear regression analysis showed significant relationships between seed covering structure hardness and ‘precipitation of the coldest quarter’, several morphological traits [area, perimeter, circularity (which considers perimeter smoothness), minimum and maximum feret, aspect ratio, roundness], as well as genus age (Fig. 4). Results from phylogenetically informed (*phylolm*) regression analyses of individual predictors with seed covering hardness as the response variable revealed that morphological seed traits such as area, perimeter, circularity, minimum and maximum feret, aspect ratio, roundness and colour (lightness) were significantly associated (Fig. 5). In addition, the climate variable ‘precipitation of the driest quarter’ was significantly negatively associated with seed covering hardness for 65 of the 100 phylogenetic trees used in the test (Fig. 5). Precipitation seasonality was also significant for two of the 100 trees, but the majority showed *P*-values just above 0.05 (Fig. 5). The remaining morphological and climatic variables, as well as genus age overall, were not significant (Fig. 5). However, genus age in relation to seed covering structure hardness of angiosperms dispersed as seeds only was nearly significant (*P*-value interval for 100 trees: 0.053–0.056). When seed covering structure hardness was used as the predictor, the results were significant for seed germination rate (positive relationship), but not for germination decline during *ex situ* storage and species distribution/range size (Fig. 5). The differences in significant results between Figs 4 and 5 show that phylogenetic relationships do seem to affect some of the variables tested in this study.

**Fig. 6.**
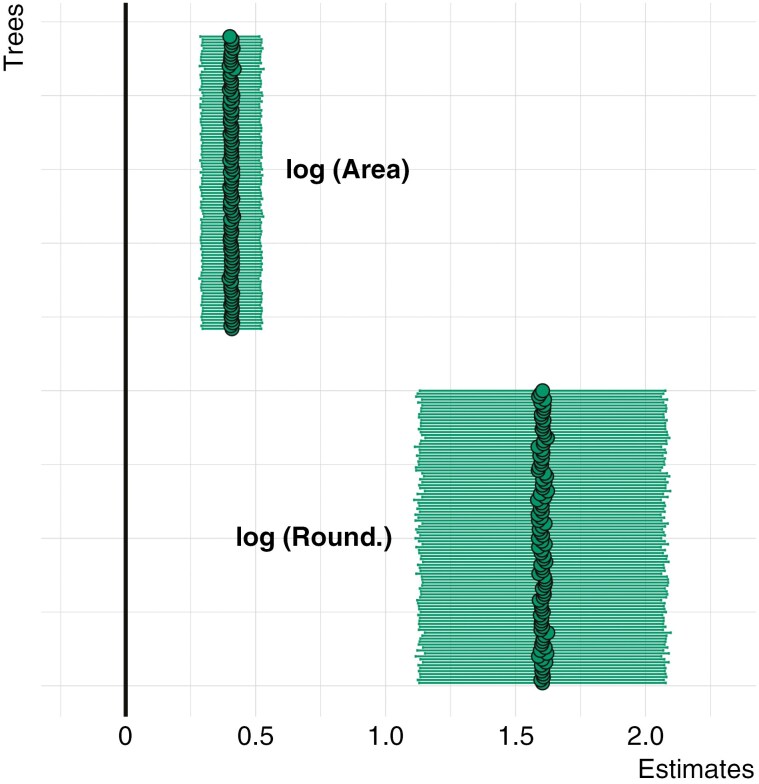
Summary of estimates and confidence intervals of phylogenetically informed minimal general linear model (*phylolm*) for *log*-transformed non-colinear climate and morphology variables against *log*-transformed seed covering structure hardness for each of the 100 phylogenetic trees by Forest (2023). Aspect ratio (1/round) was replaced with roundness for discussion purposes. All estimates are significant (*P* < 0.05).

We also performed a *phylolm* analysis with multiple climatic and morphological variables as predictors and seed covering hardness as the response variable. Of the 30 variables (Fig. 5) obtained to describe seed morphology and climatic conditions for each of the 392 taxa (Table 1), 12 were non-colinear (for correlations see Supplementary Data Fig. S2). The best minimal model (based on AIC) to explain the effect of these variables on seed covering structure hardness shows seed area (feret) and roundness (1/aspect ratio) having a significant impact, with roundness impacting the most (Fig. 6; Supplementary Data file 7).

**Fig. 7.**
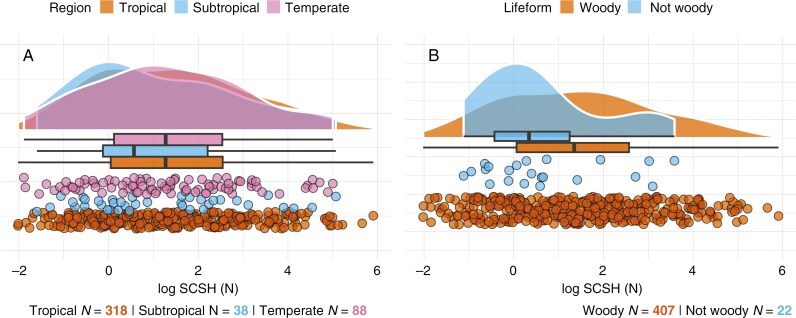
Graphs showing geographical regions and lifeforms in relation to seed covering structure hardness (SCSH) (*log*): (A) geographical regions; and (B) lifeforms. Differences between groups were tested using a Wilcoxon rank-sum/Mann–Whitney *U* test (when only two groups were present) or Kruskal–Wallis followed by Wilcoxon rank-sum/Mann–Whitney *U* test and Holm correction.

**Fig. 8.**
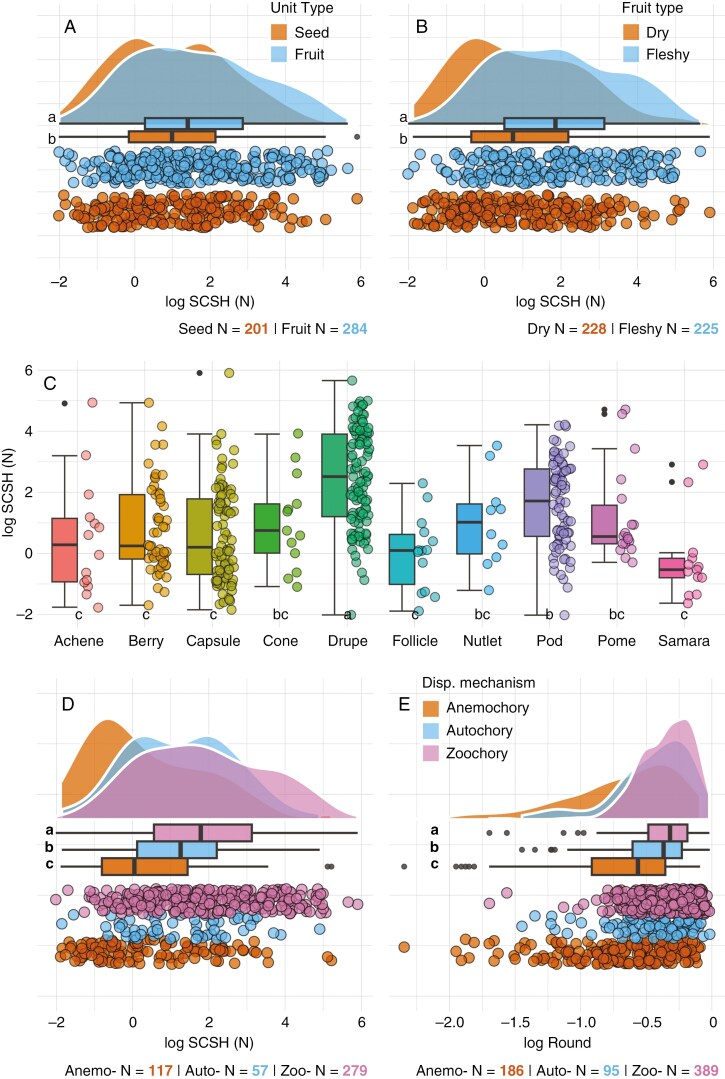
Graphs showing seed dispersal traits in relation to seed covering structure hardness (SCSH) (*log*): (A) dispersal unit type; (B) fruit type; (C) fruit subtype and (D) dispersal mechanism. In addition, (E) shows seed roundness in relation to dispersal mechanism. Differences between groups were tested using a Wilcoxon rank-sum/Mann–Whitney *U* test (when only two groups were present) or Kruskal–Wallis followed by Wilcoxon rank-sum/Mann–Whitney *U* test and Holm correction. Holm correction changed the significance grouping of several of the fruit subtypes (C).

In addition, we compared covering structure hardness across different groups of categorical variables. We found that geographical regions (tropical or temperate) and plant lifeform (woody or non woody) showed no significant differences (Fig. 7). However, hardness differed significantly between seed dormancy types (PY, MD, PD > ND) (Fig. 3D), dispersal units (fruit > seed), fruit types (fleshy > dry, drupes > other types) and dispersal mechanisms (zoochory > autochory > anemochory) (Fig. 8A–D). In addition, seed roundness was found to vary significantly between dispersal mechanisms (zoochory > autochory > anemochory) (Fig. 8E). Correcting for multiple testing of hypotheses with more than two groups did not change the significance outcomes except for several of the fruit subtypes (Fig. 8C).

Finally, one of the species measured for covering structure hardness showed within-seed differences in seed coat colour, with each seed coat characterized by a red and a black region [*Abrus precatorius* (Fabaceae)]. The red and black areas of the coat required significantly different forces for puncturing, with the black areas being ~2.4 times harder than the red areas (Fig. S5: Supplementary Data file 4).

## DISCUSSION

### Seed covering structure hardness

Our results showed that the covering structure hardness values of orthodox seeds of woody species are right/positively skewed, and only a handful of genera produce extremely hard structures (Fig. 2B; Supplementary Data file 5). As explained in ‘Variation in seed covering structure hardness’ above, seed covering structures were measured separately from other seed tissues to avoid (1) variation due to the hardness of the tissues underlying the covering structures (Frączek *et al.*, 2005) and (2) errors in estimating which part of the whole seed puncture force graph corresponds to the covering structure. Few other studies to date have measured covering structure hardness of dry seeds using direct measurements of separated structures. For example, *Lepidium didymum* fruit valve (mechanical dormancy) hardness was found to range from ~1.0 to 2.6 N depending on the area measured (Sperber *et al.*, 2017).

### Physical dormancy does not equal hardseededness

Physically dormant Fabaceae species showed higher seed covering structure hardness on average, although this difference was not significant when analysed in isolation from our overall dormancy dataset that included species from a range of families (see ‘Seed dormancy’ below). Our results for Fabaceae show a large overlap of seed coat hardness values between groups of predominantly impermeable and permeable Fabaceae species (Fig. 3A–C). In other words, permeable seed coats may be harder than impermeable coats when comparing particular species. However, it is possible that this may be different within species or for cell layers within the coat. For example, in soybean, the condition of physical dormancy can at least partially be attributed to a cuticular structure that is resistant to cracking (Ma *et al.*, 2004). In addition, several mutations have been identified in Class II KNOX genes that lead to impaired development of the cuticle layer in (cultivated) Fabaceae species, thereby rendering the seed coats permeable to water (Chai *et al.*, 2016; Laosatit *et al.*, 2022). Furthermore, seed coat thickness was found to vary significantly between physically dormant and non-dormant pea seeds (Hradilová *et al.*, 2019). Whether these observed within-species differences in cuticle structure, cuticle development and coat thickness lead to differences in cuticle or seed coat hardness has so far not been tested. There are indications that, within species, the percentage of impermeable seeds increases with whole seed hardness. For example, when studying 51 genotypes of mung bean, the percentage of impermeable seeds was 27 % for genotypes with an average whole seed hardness breaking force of 63 N versus 8 % for those measuring 22 N (Paul *et al.*, 2019). It is unclear whether this positive relationship with whole seed hardness is due to differences in hardness of the cuticle/coat only or also potentially due to effects of differences in moisture content on the hardness of the interior seed tissues (e.g. hardness of the glassy state). The fact remains that not all harder/softer mung bean seeds are impermeable/permeable to water.

Even if future research were to show that, within species, impermeable covering structures or cuticles are harder than permeable ones for all species tested, the commonly used terms ‘hardseededness’, ‘hardseeded’ and ‘seed hardness’ would not be specific enough, as they appear to refer to the ‘whole seed’ rather than particular seed structures. We therefore suggest that these terms should no longer be used in the scientific literature as synonyms for physical dormancy. We also recommend that usage of the terms ‘hard-coat’ and ‘soft-coat’ to distinguish between seeds with impermeable and permeable coats within species should be supported by appropriate measurements of seed coat hardness.

### Seed covering structure hardness and its relationships with climate and plant/seed traits

#### Climate, geographical regions and plant lifeform.

Although several seed traits are known to vary significantly between tropical and temperate regions (Visscher *et al.*, 2022), we found no significant differences in covering structure hardness between geographical zones in this study. Similarly, Chen and Moles (2018) reported that the post-dispersal biomass ratio of protective tissue to seed reserve shows no latitudinal gradient. Regarding plant lifeform, we did not find significant differences between woody and non-woody categories, although the average seed covering structure hardness was smaller for non-woody species (Fig. 7B). Similarly, the biomass ratio of seed coat to whole seed was found to be smaller for herbaceous versus woody species following phylogenetically informed analysis (Wu *et al.*, 2024). Future studies on seed covering structure hardness could include a larger number of non-woody species to address the role of lifeform more comprehensively.

The seeds that were tested for this study are predominantly desiccation-tolerant, since they were drawn from seed bank collections stored under conditions that only orthodox seeds can withstand. Our analysis therefore does not address the effect of desiccation tolerance or sensitivity on seed covering structure hardness. Previous research has shown that desiccation-sensitive seeds, which occur more frequently in tropical rainforest environments, are characterized by a relatively low seed coat to seed mass ratio (Daws *et al.*, 2006; Wu *et al.*, 2024) that can be negatively correlated with mean monthly rainfall at mean time of seed dispersal (Hill *et al.*, 2012; Obroucheva *et al.*, 2016). The seed covering structure hardness data of our orthodox seed collections showed a negative relationship with precipitation levels in the driest quarter of the year. This suggests that in environments with lower levels of precipitation during the driest season there is a trend towards harder covering structures of orthodox seeds. In regions with irregular rainfall, the seed stage often involves diaspore heteromorphism, environmentally regulated dispersal, and extended storage periods in the canopy or in the soil (Huss and Gierlinger, 2021). In addition, seed dormancy, which showed significantly harder covering structures in this study compared to non-dormancy, is associated with seasonal precipitation fluctuations (Rosbakh *et al.*, 2023), in particular in woody species (Zhang *et al.*, 2022). We could hypothesize that a well-defined dry season (regarding amount of precipitation) requires higher levels of seed covering structure hardness to survive (dormant) time periods prior to germination. Our findings regarding climate are in line with Siepielski *et al.* (2017) who showed that aspects of precipitation, rather than temperature, predicted global variation in natural selection across plant and animal populations.

#### Seed/fruit morphology: size, roundness and colour.

In an analysis of *Rubus* species, seed coat thickness was correlated with both seed weight (indicator of size) and coat hardness (Wada and Reed, 2011), suggesting that there is also a positive relationship between seed size and coat hardness. In this study, we have indeed found a positive relationship between seed/fruit size and covering structure hardness, which may be partly due to the potential for thicker coats in larger seeds. Although we found this relationship for absolute hardness (and therefore potentially absolute thickness), it is known that relative thickness (the biomass ratio of protective tissue to diaspore) did not vary consistently with sample mass (indicator of size) for 70 tropical forest species analysed by Fricke and Wright (2016).

Our results also showed a significant positive relationship between covering structure hardness and seed/fruit roundness. Seed roundness appears to play a role in seed dispersal strategies. For example, when studying 13 angiosperm species from aquatic and terrestrial habitats representing nine families, Lovas-Kiss *et al.* (2020) revealed a positive partial effect of seed roundness on seed survival through the gut passage of mallards (*Anas platyrhynchos*). In addition, Huss *et al.* (2020) compared the microstructure and geometry of different animal-dispersed plant shells and discovered that spherical shape increases the fracture resistance via geometric stiffening. They conclude that geometry is particularly important to resist shell cracking by granivores and that spherical shells are more advantageous than elongated shells of similar size and thickness due to a higher rigidity (Huss and Gierlinger, 2021). Indeed, our analysis confirmed that seeds and fruits with evidence for animal dispersal (zoochory) are characterized by a significantly higher degree of roundness on average than seeds and fruits that are dispersed through other methods (autochory, anemochory).

Regarding colour we found that lightness has a positive relationship with covering structure hardness. Seed and fruit coats of lighter colour were generally shown to be harder. This was contrary to our expectations based on findings within particular species (Prasad and Weigle, 1976; Mohamed-Yasseen *et al.*, 1994). In addition, we discovered a significant within-seed difference in coat hardness (black > red) in a Fabaceae species with a bicolour coat. However, this difference could have been caused by (bio)chemical and (bio)physical factors unrelated to those causing the variation in colour.

#### Seed dormancy.

We found that in our overall data set based on species from a range of families, seeds with PY were characterized on average by significantly harder covering structures than non-dormant seeds. However, all dormancy types were significantly different from non-dormant seeds, with no significant differences between dormancy types (PY, MD, PD). In addition, as seen for Fabaceae, there was large overlap between the seed covering structure hardness values of the different seed dormancy categories, meaning that certain permeable covering structures are harder than particular impermeable structures (Fig. 3D). These results further confirm the conclusion drawn from the Fabaceae case study that (variations of) the terms ‘hardseeded’ or ‘hard coat’ should not be used as synonyms for physical dormancy in general. As discussed above, seed dormancy and seed covering structure hardness appear to have a positive relationship with irregular and low dry season precipitation respectively (see ‘Seed/fruit morphology: size, roundness and colour’ above).

#### Seed germination rate and longevity.

Seed covering structure hardness was shown to have a positive relationship with germination rate (seeds with harder structures take longer to germinate). We could hypothesize that this is at least partially due to the observation that seeds with dormancy had significantly harder covering structures on average, although the germination test data used for this analysis may have included tests with artificial dormancy breaking treatments.

Our research did not show a relationship between seed covering structure hardness and germination decline during storage (*ex situ* seed longevity). Although we found that physically dormant (water-impermeable) seeds were on average harder than water-permeable (non-dormant) seeds, we have no information available on permeability to oxygen, which is known to reduce seed longevity under *ex situ* storage conditions (versus anoxia), particularly for glassy cytoplasm (Groot *et al.*, 2015; Gerna *et al.*, 2022). This result does not rule out the possibility that hardness can predict seed longevity *in situ*. As mentioned before, coat thickness and hardness correlated with longevity of seeds in soil environments (Hill and Vander Kloet, 2005; Gardarin *et al.*, 2010; Schutte *et al.*, 2014).

#### Seed dispersal and species distribution/range size.

We found significantly harder covering structures for several seed traits linked to seed dispersal type and mechanism. For example, seeds dispersed as fruit showed harder covering structures than seeds dispersed on their own. Covering structures from species with fleshy fruits were harder than those with dry fruits and species with drupes had the hardest covering structures compared to species with other fruit types. Regarding dispersal mechanism, we found that covering structures from seeds and fruits dispersed by animals were significantly harder and rounder than those of seeds and fruits dispersed by wind. In an analysis of fruit evolution, Lorts *et al.* (2008) found no clear association between fruit types and major angiosperm lineages and concluded that fruit evolution was driven at least in part by dispersal agents abundant in particular habitats.

Although we found a significant positive relationship between covering structure hardness and seed/fruit size, and despite previous research showing that small-seeded species disperse further and tend to have larger ranges than large-seeded species (Morin and Chuing, 2006; Thomson *et al.*, 2011), our analysis revealed no overall significant effect of seed covering structure hardness on species distribution range size. However, extremely large distributions seem to be restricted to seeds with lower hardness values, whilst the hardest values appear to be linked to having smaller distributions (Fig. S6: Supplementary Data file 4).

#### Genus age.

Our results showed an overall tendency (*phylolm* estimate [slope]: 0.11) that older genera have harder seed covering structures, although this was non-significant (*P*-value interval: 0.32–0.35). Whilst existing literature does not address the evolution of covering structure hardness directly, there are indications that hardness is related to thickness for some species (Blate *et al.*, 1998; Wada and Reed, 2011). Seed coat thickness is determined by both integument number and cell thickness (Coen and Magnani, 2018). Extant gymnosperm ovules are typically unitegmic (covered by one integument) whilst the ancestral state of angiosperm ovules is bitegmic, with unitegmy arising several times during their evolutionary history (Lora *et al.*, 2015; Coen and Magnani, 2018; Rudall, 2021). In the current study, 97 % of the seed collections tested for covering structure hardness were from angiosperms and at least 44 % are dispersed as seeds. When limiting our analysis to angiosperms dispersed as seeds (focusing on seed coats only), the *phylolm* estimate [slope] increased to 0.27 and the *P*-values decreased to 0.053–0.056. This suggests that, aside from time for evolutionary adaptation, there is a possibility that the number of integuments might play a role in the tendency of older genera to show a trend towards harder covering structures. Future research could directly analyse seed covering structure hardness in relation to integument number.

### Research limitations

Our research has some limitations which could be overcome by further investigation. First, it was restricted in diversity (relative to all known plant species) and covered mainly woody species with predominantly desiccation-tolerant seeds. It would be valuable to expand the sampling of our study to cover recalcitrant seeds and herbaceous species to determine how widely applicable the results that we obtained are. Second, whilst this was out of scope for our study, analysing the (bio)chemical/molecular composition and (bio)physical structure (thickness, cell types, number of integuments) of seed covering structures may be important to further understand the mechanisms underlying variation in hardness. Finally, we did not address the effect of fire variables on covering structure hardness or the effect of covering structure hardness on seed persistence in the soil seed bank, but further investigations in these areas would provide valuable information for deciphering the evolutionary processes that led to the survival of species under potentially harsh environments.

## CONCLUSIONS

This first global assessment of seed covering structure hardness can serve as a foundation for future studies. Our results (alpha level set at 0.05) suggest roles for seed morphology, dispersal, dormancy and precipitation in explaining part of the global variation (by more than 2000-fold) in seed covering structure hardness of woody species with orthodox seeds. Morphological traits such as seed size and roundness showed highly significant positive associations, both in individual and combined phylogenetically informed regression analyses. Harder covering structures were also shown to occur in dormant seeds/fruits and areas with lower precipitation during the driest quarter (e.g. areas with a well-defined dry season), with dormancy possibly playing a role in slowing germination rate. Importantly, we showed that PY is not always synonymous with having a harder covering structure. Harder structures were also found in seeds dispersed as fruits and in species with seeds carried in fleshy fruits. Both covering structure hardness and roundness were higher in animal-dispersed seeds/fruits compared to units with other main dispersal strategies. Of all the variables we initially identified with potential to explain seed covering structure hardness, many did not show significant associations. These include the majority (18/19) of the climate (precipitation and temperature) variables, elevation, two of the three colour variables, genus age, geographical region and plant lifeform.

Regarding seed covering structure hardness as a potential predictor of other traits, we found that it predicts germination rate but not *ex situ* seed longevity or species distribution, although we observed some limitations at the extremes of the range sizes.

## Supplementary Data

Supplementary data are available at *Annals of Botany* online and consist of the following.

File 1: Data on seed covering structure hardness, taxonomy, climate, elevation, plant lifeform, seed dispersal, seed dormancy, *ex situ* seed longevity (dRG), seed germination rate, seed morphology, seed colour, species distribution [range size (EOO)], Fabaceae seed imbibition tests.

File 2: Fabaceae seed imbibition raw data.

File 3: Dispersal data sources.

File 4: Figures S1, S2, S3, S5, S6.

File 5: Seed collections that could not be measured for seed covering structure hardness.

File 6: Figure S4; family-level phylogenetic tree with average (log) SCSH values per family.

File 7: Table with results of phylogenetically informed minimal general linear model using *phylolm*.

mcaf027_suppl_Supplementary_Files_1

mcaf027_suppl_Supplementary_Files_6_Figures_S4

mcaf027_suppl_Supplementary_Files_2

mcaf027_suppl_Supplementary_Files_3

mcaf027_suppl_Supplementary_Files_4_Figures_S1-S6

mcaf027_suppl_Supplementary_Files_5

mcaf027_suppl_Supplementary_Files_7

## Data Availability

The data and code used to replicate the analyses are available on Figshare (https://doi.org/10.6084/m9.figshare.25941184). Phylogenetic trees used in this study are not included in this repository but can be downloaded from Forest (2023).

## Author Declarations

The authors contributed to the manuscript in the following manner: conception and design (AMV, HWP); acquisition of data (AMV, PGB, UL, YW, DM, AC, SM); analysis and interpretation of the data (AMV, PGB, MSG, AC, UL, YW, FF); drafting of the article (AMV, PGB, UL, MSG, AC, YW, HWP); critical revision of the article for important intellectual content (AMV, PGB, MSG, AC, UL, YW, DM, FF, SM, HWP); final approval of the article (AMV, PGB, MSG, AC, UL, YW, DM, FF, SM, HWP). AMV (a.visscher@kew.org) and PGB (p.gomezbarreiro@kew.org) take responsibility for the integrity of the research from inception to finished article.
