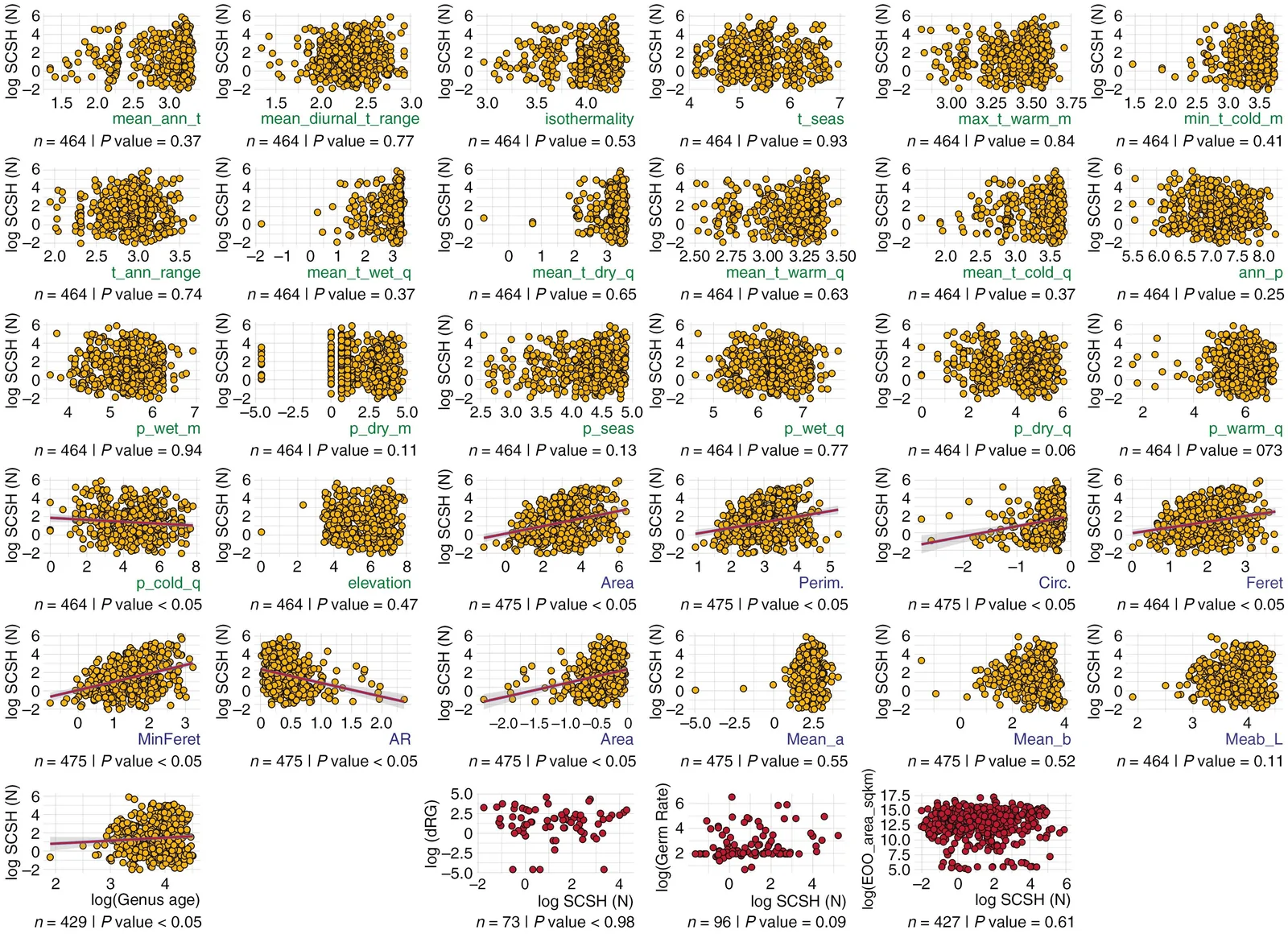# Correction to: Global variation in seed covering structure hardness of woody species with orthodox seeds

**DOI:** 10.1093/aob/mcag221

**Published:** 2026-07-29

This is a correction to: Anne M Visscher, Pablo Gómez Barreiro, Marybel Soto Gomez, Angelino Carta, Udayangani Liu, Yu Wu, Deshika Muthuthanthirige, Félix Forest, Sian McCabe, Hugh W Pritchard, Global variation in seed covering structure hardness of woody species with orthodox seeds, Annals of Botany, Volume 136, Issue 2, July 2025, Pages 419–436, https://doi.org/10.1093/aob/mcaf027

In the originally published version of this manuscript, the labels of Figure 3 and Figure 4 contained errors introduced during production.

In Figure 3D, group labels at the top of the panel have been corrected to read: “ND=41, PY=29, PD=60, MD=17” instead of: “ND=41, PY=29, PY=60, PY=17”.

The corrected Figure 3 reads:

**Figure:**
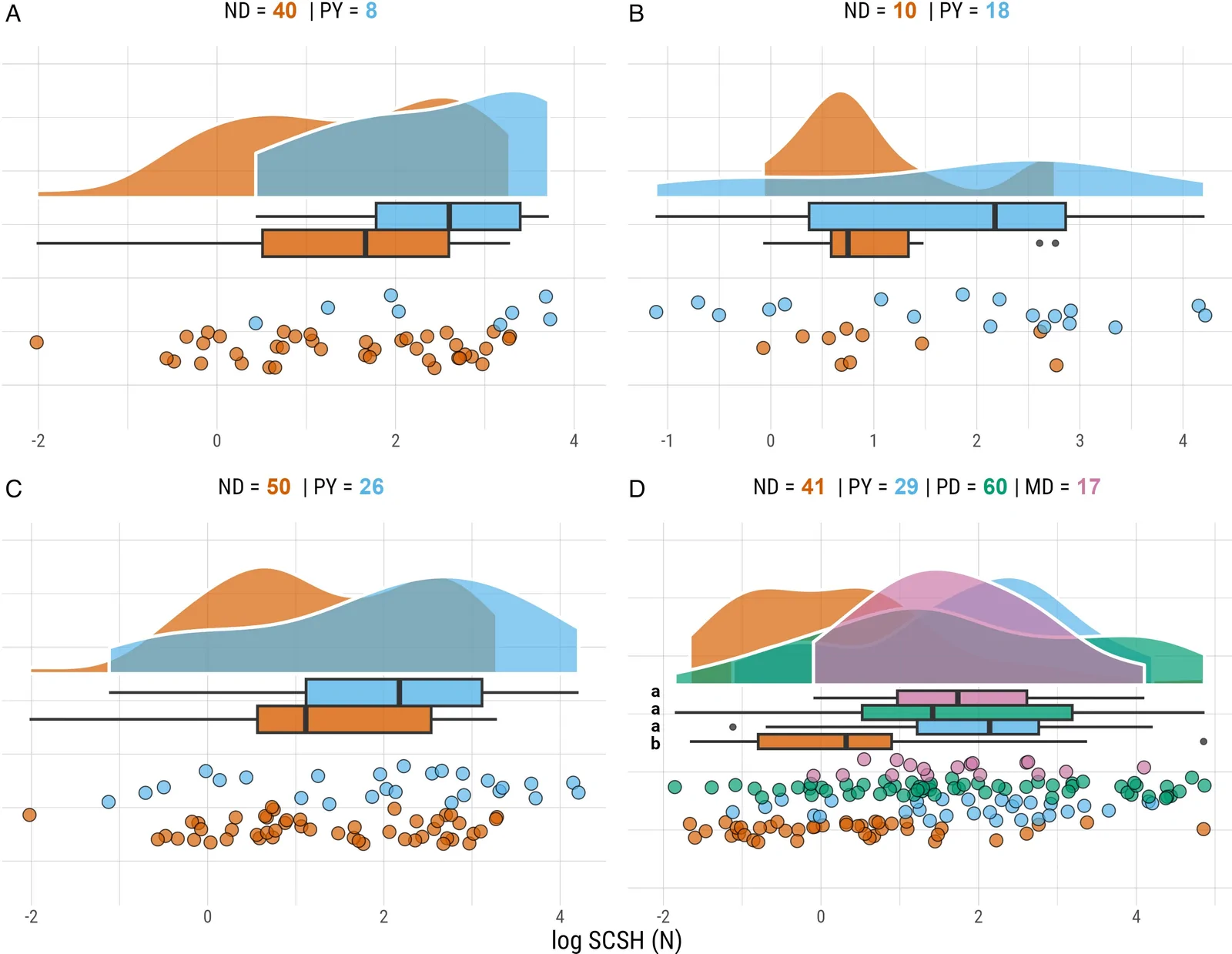


instead of:

**Figure:**
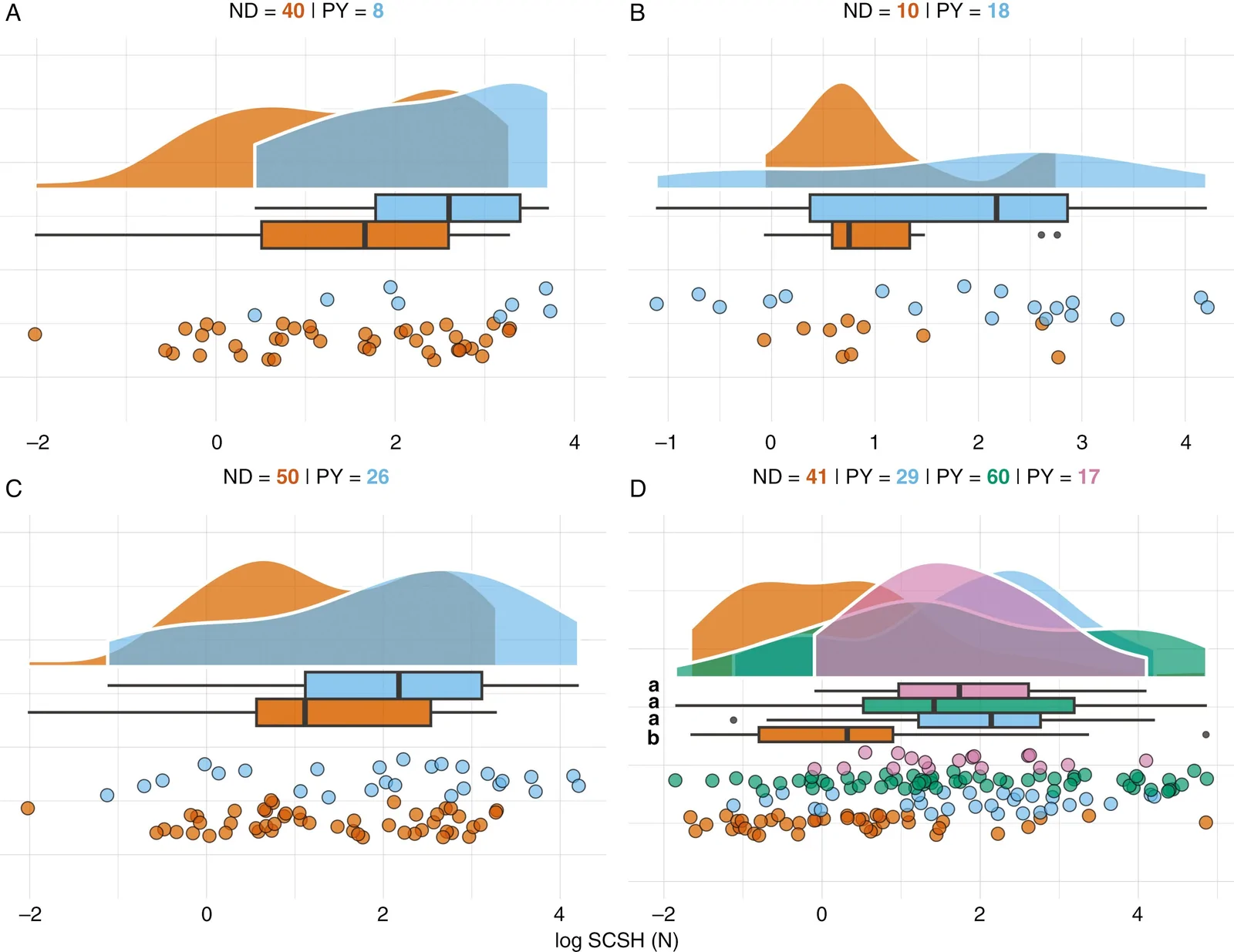


In Figure 4, a panel has been corrected to read “Round” underneath the horizontal x-axis instead of “Area”. Another panel has been corrected to read “Mean_L” instead of “Meab_L”. An additional panel labelled log(dRG) along the vertical y-axis has been corrected to read “p value = 0.98” underneath the x axis instead of “p value < 0.98”.

The corrected Figure 4 reads:

**Figure:**
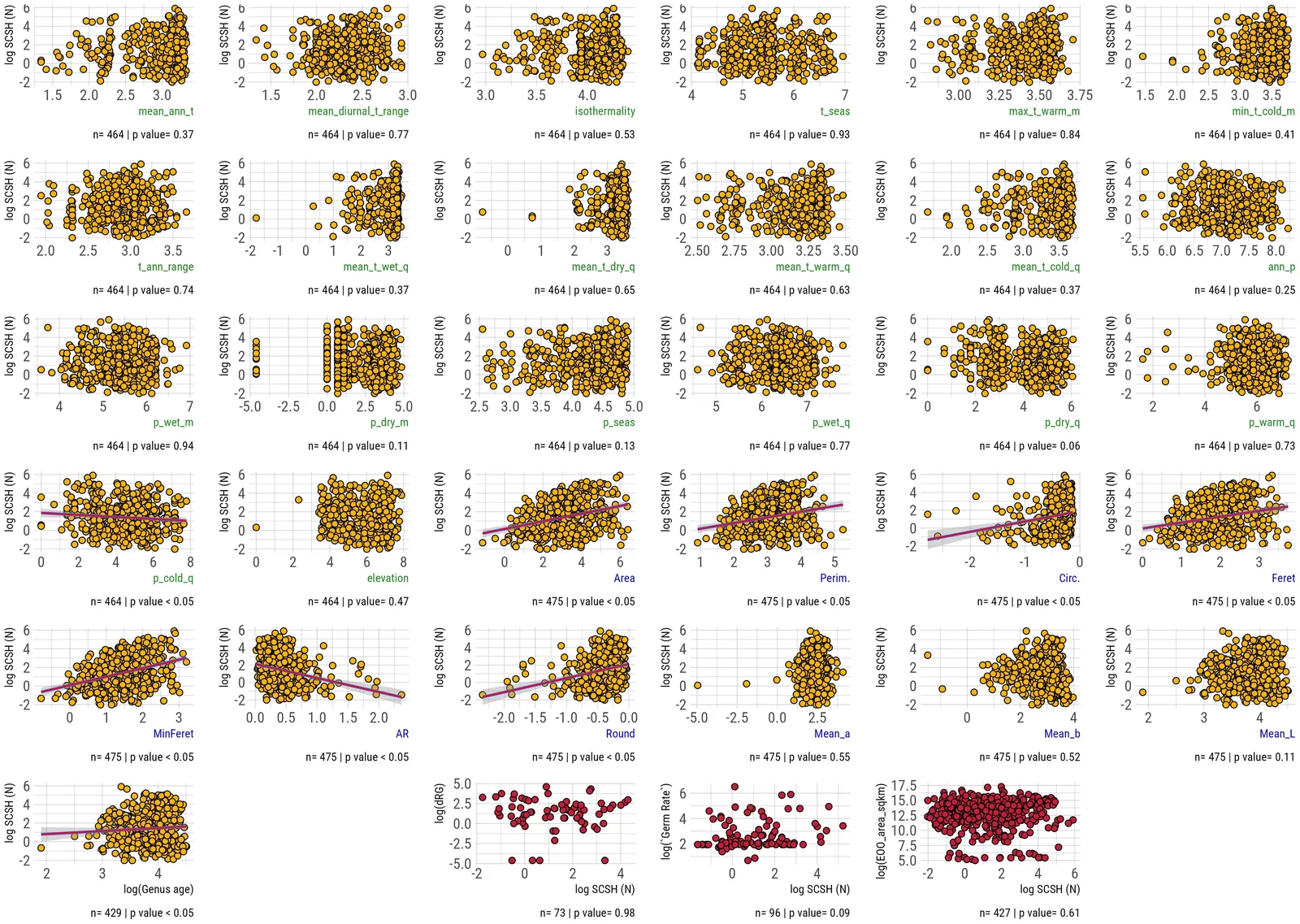


instead of:

**Figure:**