# Supplementary material for: Global variation in seed covering structure hardness of woody species with orthodox seeds
Source: Ann Bot. 2025 Feb 27;136(2):419–36. doi: 10.1093/aob/mcaf027 (PMC12445846; doi:10.1093/aob/mcaf027)
Supplement: mcaf027_suppl_Supplementary_Files_6_Figures_S4 [file mcaf027_suppl_supplementary_files_6_figures_s4.pdf]

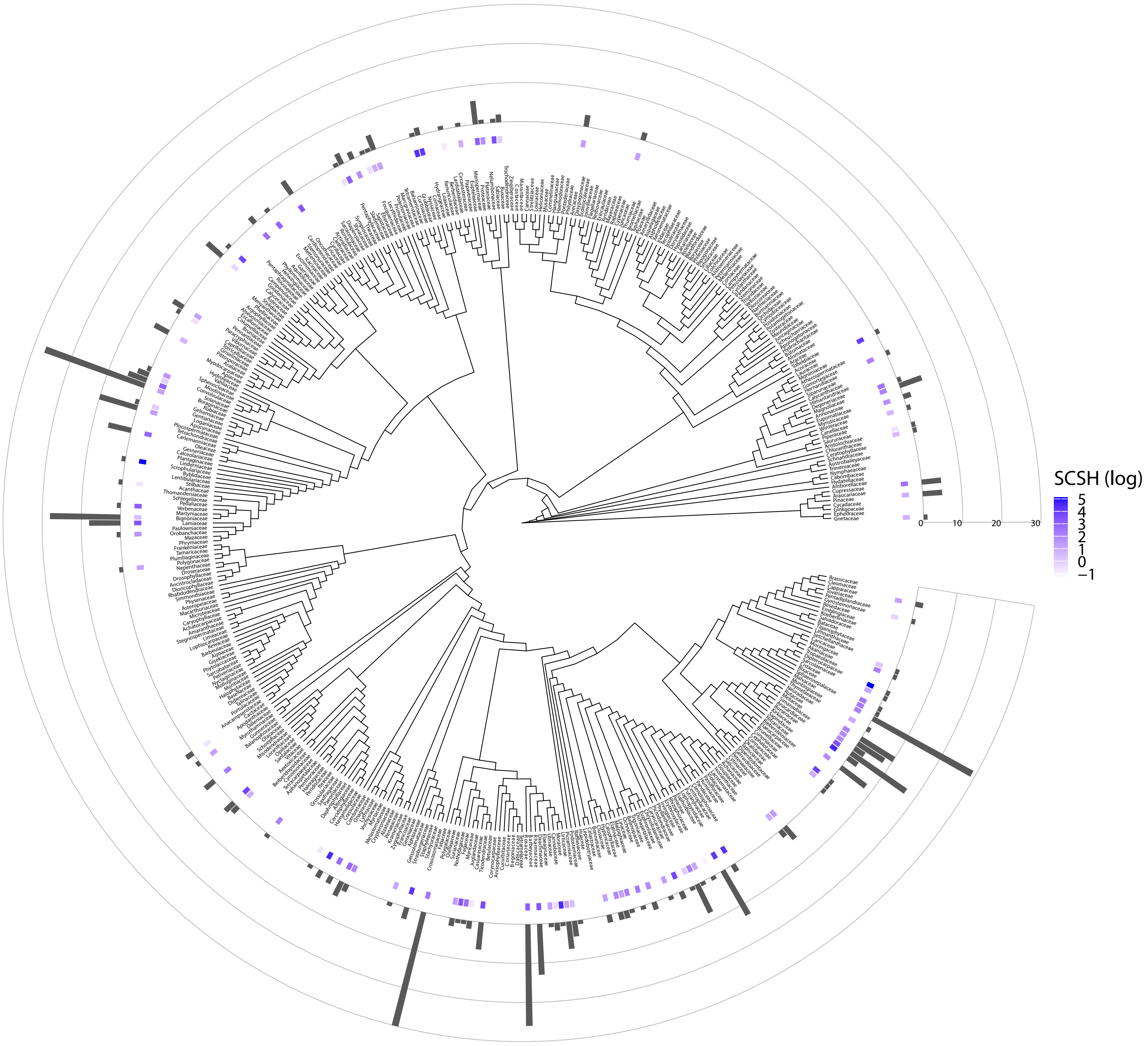

Fig. S4. Family-level phylogenetic tree with families sampled for this study indicated by bars representing the number of species sampled and boxes representing the average (log) SCSH values of species sampled. Note that 88 species of Fabaceae were sampled, but that the family is here represented with a lower number (i.e. 30) to accentuate the representation of other families
