# Supplementary material for: Global variation in seed covering structure hardness of woody species with orthodox seeds
Source: Ann Bot. 2025 Feb 27;136(2):419–36. doi: 10.1093/aob/mcaf027 (PMC12445846; doi:10.1093/aob/mcaf027)
Supplement: mcaf027_suppl_Supplementary_Files_4_Figures_S1-S6 [file mcaf027_suppl_supplementary_files_4_figures_s1-s6.docx]

**Supplementary Figures S1, S2, S3, S5, S6**

**Supplementary Figure S1**


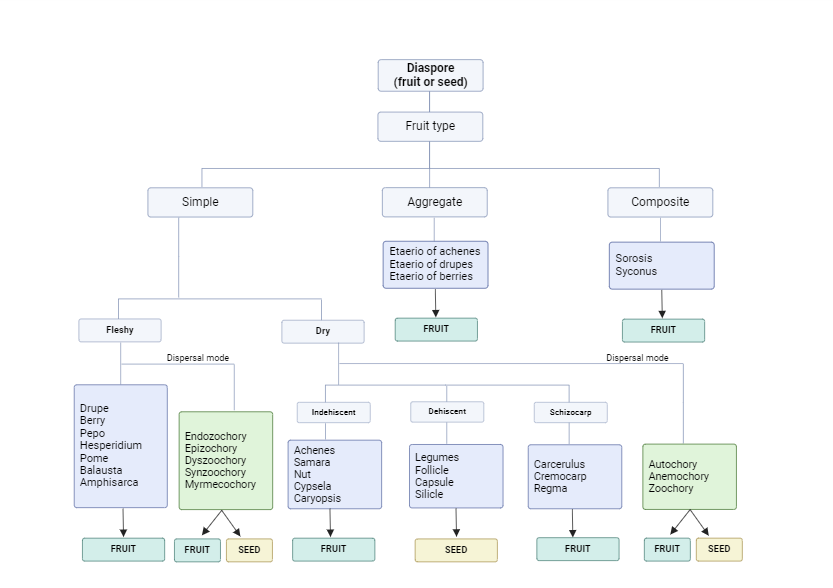


Diagram showing the process of determining: 1) whether a species is dispersed as a seed only or as a seed in fruit tissues, 2) whether the seed was/is contained in a dry or fleshy fruit, 3) the type of fruit that the seed was/is contained in.

**Supplementary Figure S2**

**
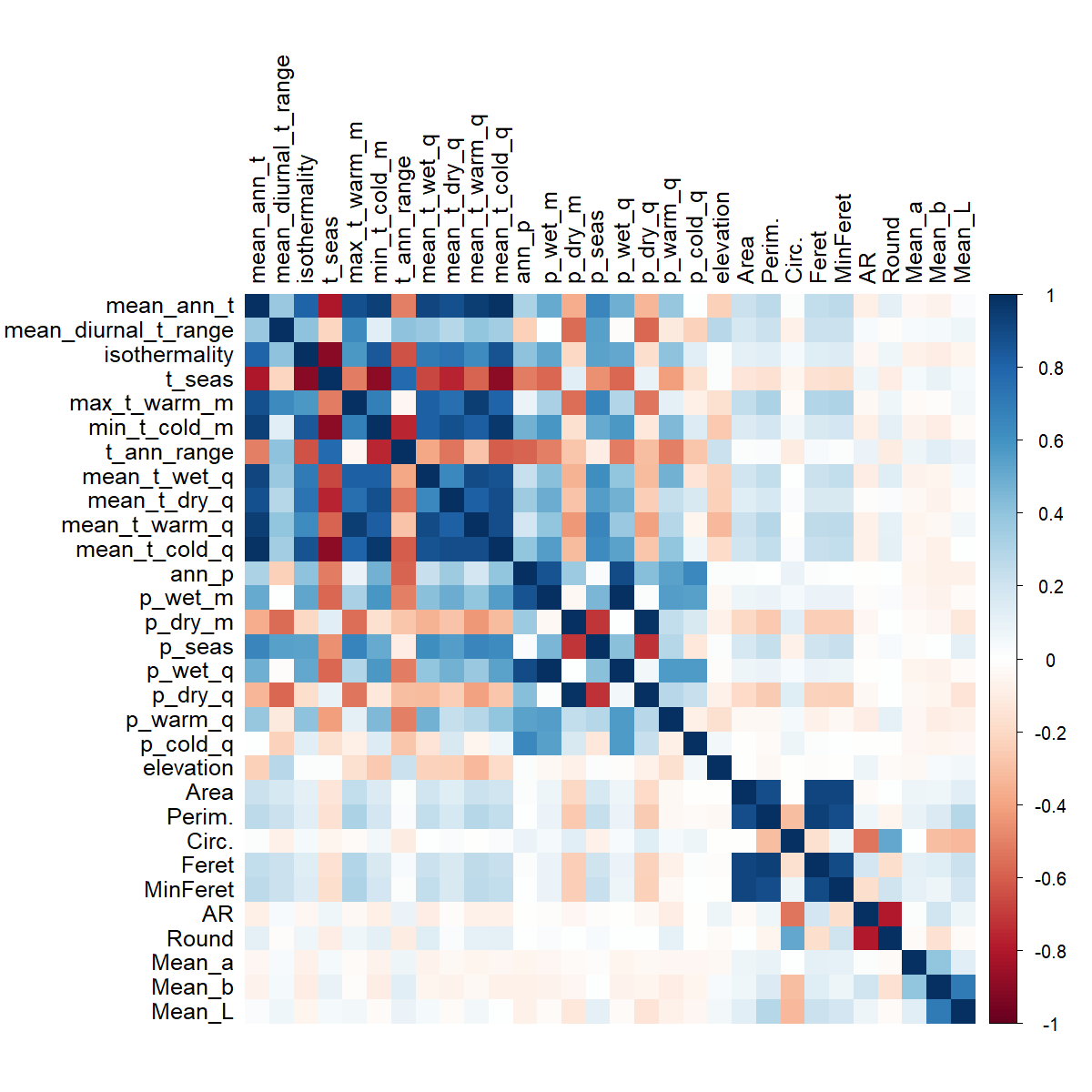
**

Graph showing a correlation matrix of continuous climate, elevation and morphological variables.

**Supplementary Figure S3**

**
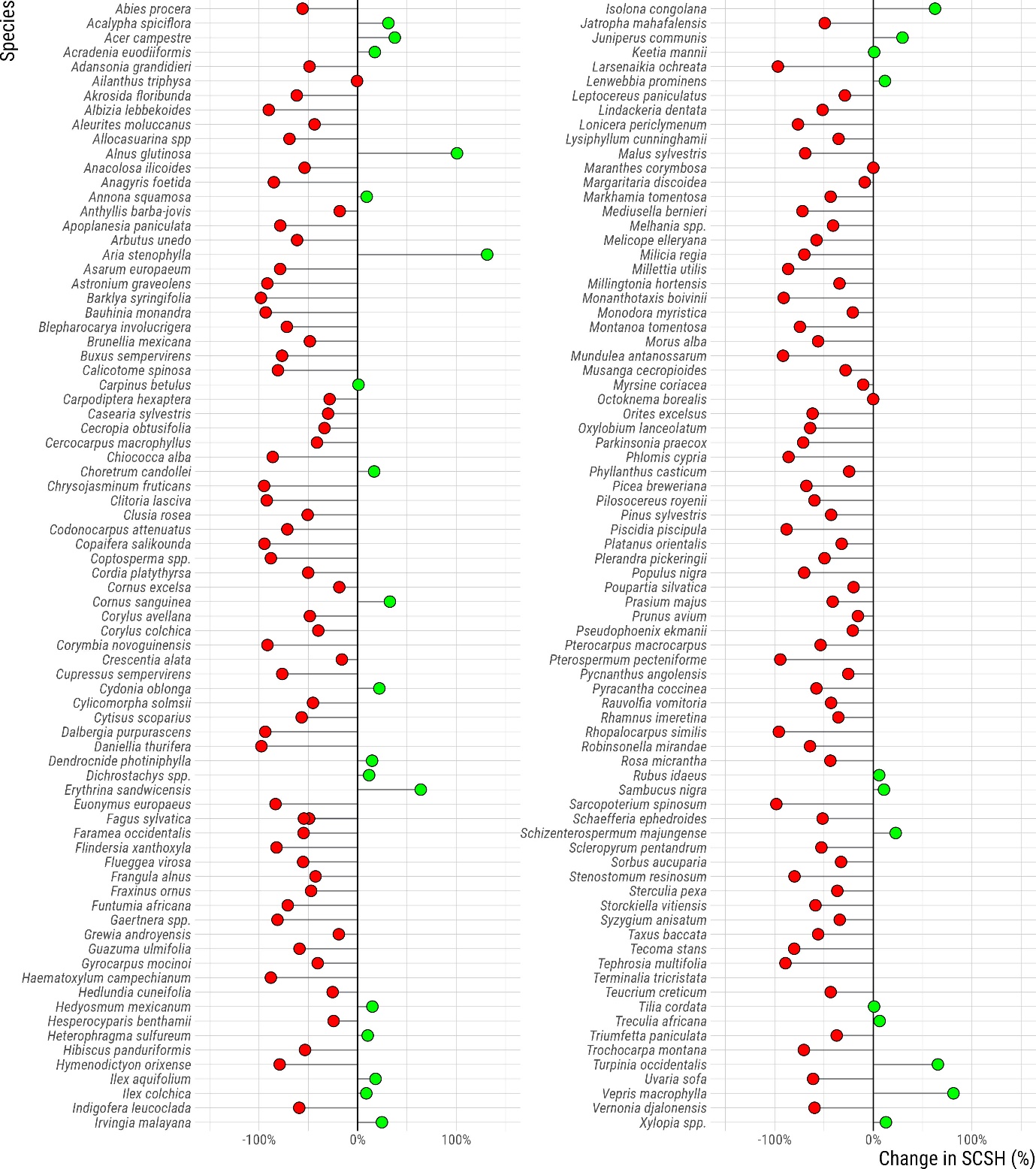
**

Figure showing percentage decrease (red) or increase (green) of seed covering structure hardness (SCSH) after removing seed internal structures relative to the average SCSH of an intact seed. *Terminalia tricristata* is not shown in the graph (3800% increase in the required force).

**Supplementary Figure S5**


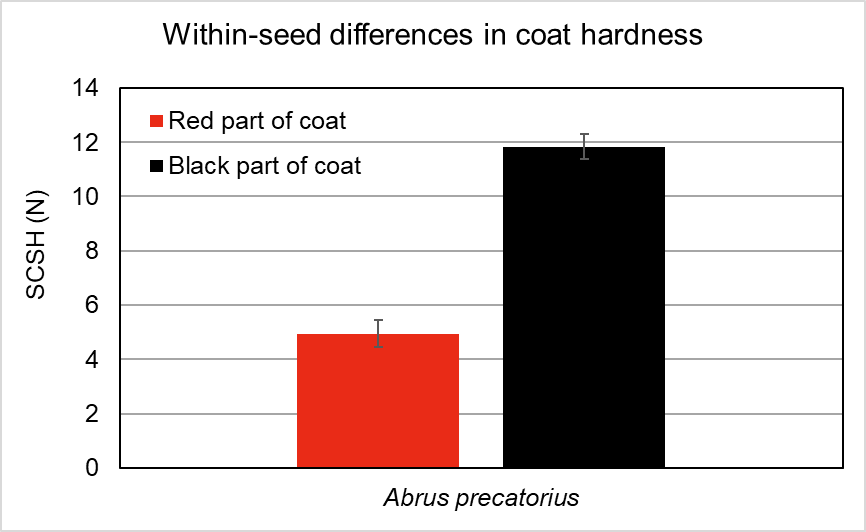


Graph showing within-seed differences in seed covering structure hardness (SCSH) of red and black coat regions of *Abrus precatorius* (Fabaceae). The red and black areas of the coat required significantly different forces for puncturing (t-test in Excel), with the black areas being approximately 2.4 times harder than the red areas.

**Supplementary Figure S6**


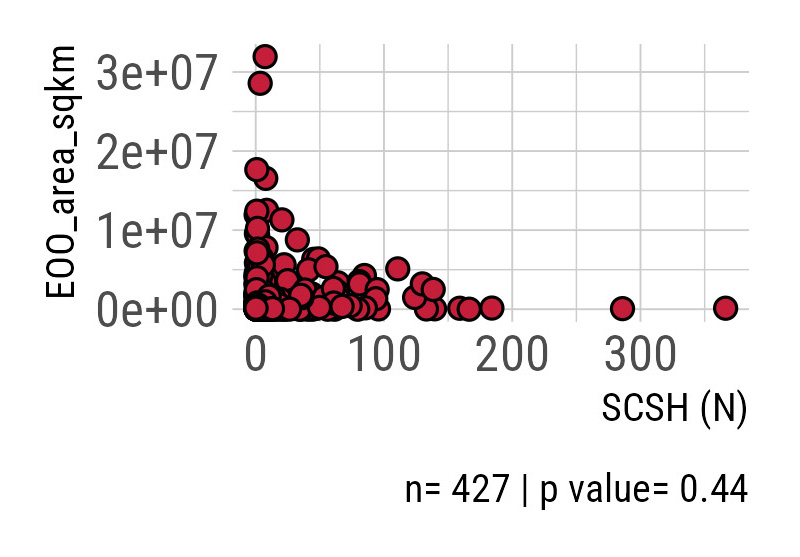


Graph showing untransformed seed covering structure hardness (SCSH) (N) versus species distribution (range). Extremely large distributions seem to be restricted to seeds with lower covering structure hardness values, whilst the hardest values appear to be linked to species having smaller distributions.
