# Supplementary material for: Global variation in seed covering structure hardness of woody species with orthodox seeds
Source: Ann Bot. 2025 Feb 27;136(2):419–36. doi: 10.1093/aob/mcaf027 (PMC12445846; doi:10.1093/aob/mcaf027)
Supplement: mcaf027_suppl_Supplementary_Files_7 [file mcaf027_suppl_supplementary_files_7.pdf]

Phylolm result for *log*(SCSH)~minimal model, all non colinear

tree_n	ultrametic	bifurcating	variables	aic	lambda	sigma2	r_sq
1	TRUE	TRUE	log(AR)+log(Area)	1433.487	0.4849352	2.745338	0.2033171
2	TRUE	TRUE	log(AR)+log(Area)	1435.314	0.4630485	2.707243	0.1994052
3	TRUE	TRUE	log(AR)+log(Area)	1432.693	0.5056714	2.787849	0.2050739
4	TRUE	TRUE	log(AR)+log(Area)	1431.104	0.5419578	2.861718	0.2026091
5	TRUE	TRUE	log(AR)+log(Area)	1431.332	0.5268199	2.827246	0.2009081
6	TRUE	TRUE	log(AR)+log(Area)	1431.682	0.5448319	2.878046	0.2042310
7	TRUE	TRUE	log(AR)+log(Area)	1433.701	0.4787598	2.733417	0.2027109
8	TRUE	TRUE	log(AR)+log(Area)	1431.437	0.4999131	2.765073	0.2064997
9	TRUE	TRUE	log(AR)+log(Area)	1430.071	0.5419512	2.855685	0.2037606
10	TRUE	TRUE	log(AR)+log(Area)	1433.100	0.4797003	2.730233	0.2009971
11	TRUE	TRUE	log(AR)+log(Area)	1428.536	0.5897196	2.977105	0.2062756
12	TRUE	TRUE	log(AR)+log(Area)	1432.366	0.5049778	2.780820	0.2019743
13	TRUE	TRUE	log(AR)+log(Area)	1428.881	0.5578998	2.888882	0.2076236
14	TRUE	TRUE	log(AR)+log(Area)	1434.221	0.4907179	2.762328	0.2004128
15	TRUE	TRUE	log(AR)+log(Area)	1432.083	0.5084025	2.788496	0.2004571
16	TRUE	TRUE	log(AR)+log(Area)	1433.381	0.5062158	2.791574	0.2023656
17	TRUE	TRUE	log(AR)+log(Area)	1430.987	0.5189085	2.806751	0.2054092
18	TRUE	TRUE	log(AR)+log(Area)	1431.522	0.5411203	2.866682	0.2005952
19	TRUE	TRUE	log(AR)+log(Area)	1430.989	0.5333581	2.839036	0.2028692
20	TRUE	TRUE	log(AR)+log(Area)	1432.913	0.4906097	2.755155	0.2024918
21	TRUE	TRUE	log(AR)+log(Area)	1431.585	0.5189238	2.808897	0.2054766
22	TRUE	TRUE	log(AR)+log(Area)	1431.197	0.5231305	2.817425	0.2034481
23	TRUE	TRUE	log(AR)+log(Area)	1430.526	0.5165668	2.797214	0.2063229
24	TRUE	TRUE	log(AR)+log(Area)	1431.260	0.5362197	2.851229	0.2033436
25	TRUE	TRUE	log(AR)+log(Area)	1430.845	0.5343144	2.843719	0.2059286
26	TRUE	TRUE	log(AR)+log(Area)	1431.972	0.5112488	2.794760	0.2029563
27	TRUE	TRUE	log(AR)+log(Area)	1430.490	0.5340269	2.839461	0.2042389
28	TRUE	TRUE	log(AR)+log(Area)	1435.710	0.4629751	2.711223	0.2009265
29	TRUE	TRUE	log(AR)+log(Area)	1430.516	0.5271491	2.820702	0.2018673
30	TRUE	TRUE	log(AR)+log(Area)	1432.080	0.5339662	2.854329	0.2016659
31	TRUE	TRUE	log(AR)+log(Area)	1434.893	0.4678916	2.715644	0.2020845
32	TRUE	TRUE	log(AR)+log(Area)	1433.010	0.5330348	2.855871	0.2014473
33	TRUE	TRUE	log(AR)+log(Area)	1430.549	0.5591181	2.904389	0.2014564
34	TRUE	TRUE	log(AR)+log(Area)	1430.582	0.5361365	2.847340	0.2044317
35	TRUE	TRUE	log(AR)+log(Area)	1431.710	0.5323041	2.843937	0.2039345
36	TRUE	TRUE	log(AR)+log(Area)	1433.057	0.5166837	2.814149	0.2034305
37	TRUE	TRUE	log(AR)+log(Area)	1432.111	0.5129710	2.801181	0.2038497
38	TRUE	TRUE	log(AR)+log(Area)	1435.087	0.4550191	2.690313	0.1994228
39	TRUE	TRUE	log(AR)+log(Area)	1429.168	0.5549002	2.882053	0.2072097
40	TRUE	TRUE	log(AR)+log(Area)	1429.032	0.5496424	2.869742	0.2063111
41	TRUE	TRUE	log(AR)+log(Area)	1430.455	0.5348783	2.843477	0.2017162
42	TRUE	TRUE	log(AR)+log(Area)	1432.465	0.4979945	2.764374	0.2031738
43	TRUE	TRUE	log(AR)+log(Area)	1432.971	0.5171594	2.813657	0.2018202
44	TRUE	TRUE	log(AR)+log(Area)	1431.671	0.5275644	2.830571	0.2021998
45	TRUE	TRUE	log(AR)+log(Area)	1430.333	0.5369771	2.844438	0.2047671
46	TRUE	TRUE	log(AR)+log(Area)	1433.444	0.4846544	2.742476	0.2007956
47	TRUE	TRUE	log(AR)+log(Area)	1430.915	0.5372040	2.848652	0.2036236
48	TRUE	TRUE	log(AR)+log(Area)	1428.807	0.5574451	2.887655	0.2030842
49	TRUE	TRUE	log(AR)+log(Area)+log(Circ.)	1428.969	0.5464683	2.849218	0.2062262
50	TRUE	TRUE	log(AR)+log(Area)	1431.759	0.5438573	2.876270	0.2026720
51	TRUE	TRUE	log(AR)+log(Area)	1430.986	0.5259298	2.820987	0.2039680
52	TRUE	TRUE	log(AR)+log(Area)	1434.815	0.4792865	2.741471	0.2018735
53	TRUE	TRUE	log(AR)+log(Area)	1434.739	0.4737954	2.729226	0.2017167
54	TRUE	TRUE	log(AR)+log(Area)	1435.100	0.4735247	2.728688	0.1998882
55	TRUE	TRUE	log(AR)+log(Area)	1430.478	0.5256380	2.815620	0.2066559
56	TRUE	TRUE	log(AR)+log(Area)	1431.737	0.5210377	2.816650	0.2008560
57	TRUE	TRUE	log(AR)+log(Area)	1431.058	0.5294058	2.835152	0.2015249
58	TRUE	TRUE	log(AR)+log(Area)	1434.568	0.4883151	2.758747	0.1983264
59	TRUE	TRUE	log(AR)+log(Area)	1430.995	0.5416381	2.864889	0.2038401
60	TRUE	TRUE	log(AR)+log(Area)	1433.514	0.5056884	2.794305	0.2000527
61	TRUE	TRUE	log(AR)+log(Area)	1433.065	0.4798021	2.732439	0.2027177
62	TRUE	TRUE	log(AR)+log(Area)	1431.791	0.5143782	2.801253	0.2022585
63	TRUE	TRUE	log(AR)+log(Area)	1434.401	0.4758524	2.731356	0.1987611
64	TRUE	TRUE	log(AR)+log(Area)	1429.754	0.5393072	2.845507	0.2059364
65	TRUE	TRUE	log(AR)+log(Area)	1431.464	0.5250130	2.824975	0.2010972
66	TRUE	TRUE	log(AR)+log(Area)	1431.819	0.5107647	2.792589	0.2046691
67	TRUE	TRUE	log(AR)+log(Area)	1430.355	0.5488256	2.876564	0.2014495
68	TRUE	TRUE	log(AR)+log(Area)	1434.205	0.4756162	2.729150	0.2031297
69	TRUE	TRUE	log(AR)+log(Area)	1428.320	0.5598871	2.889231	0.2057088
70	TRUE	TRUE	log(AR)+log(Area)	1432.171	0.5217273	2.818373	0.2021088
71	TRUE	TRUE	log(AR)+log(Area)	1433.238	0.4830548	2.738327	0.2016970
72	TRUE	TRUE	log(AR)+log(Area)	1430.364	0.5387279	2.853405	0.2023612
73	TRUE	TRUE	log(AR)+log(Area)	1431.529	0.5260637	2.826038	0.2020203
74	TRUE	TRUE	log(AR)+log(Area)	1435.427	0.4704650	2.724987	0.1988528
75	TRUE	TRUE	log(AR)+log(Area)	1431.633	0.5158849	2.804404	0.2053292
76	TRUE	TRUE	log(AR)+log(Area)	1432.983	0.5132370	2.805442	0.1985156
77	TRUE	TRUE	log(AR)+log(Area)	1431.270	0.5391369	2.855863	0.2006392
78	TRUE	TRUE	log(AR)+log(Area)	1434.207	0.4668005	2.709210	0.2035882
79	TRUE	TRUE	log(AR)+log(Area)	1432.447	0.5369844	2.862832	0.2003992
80	TRUE	TRUE	log(AR)+log(Area)	1431.580	0.5203638	2.816801	0.2054195
81	TRUE	TRUE	log(AR)+log(Area)	1435.603	0.4714160	2.728341	0.2002140
82	TRUE	TRUE	log(AR)+log(Area)	1435.889	0.4622358	2.713494	0.1990901
83	TRUE	TRUE	log(AR)+log(Area)	1431.251	0.5194862	2.807838	0.2024987
84	TRUE	TRUE	log(AR)+log(Area)	1426.440	0.5778411	2.926966	0.2079150
85	TRUE	TRUE	log(AR)+log(Area)	1429.528	0.5451802	2.862856	0.2049766
86	TRUE	TRUE	log(AR)+log(Area)	1434.834	0.4589943	2.697354	0.2014420
87	TRUE	TRUE	log(AR)+log(Area)	1435.268	0.4671683	2.718814	0.1985280
88	TRUE	TRUE	log(AR)+log(Area)+log(Circ.)	1429.827	0.5231132	2.794347	0.2101524
89	TRUE	TRUE	log(AR)+log(Area)	1428.860	0.5364771	2.833357	0.2100516
90	TRUE	TRUE	log(AR)+log(Area)	1435.658	0.4536814	2.692244	0.1998165
91	TRUE	TRUE	log(AR)+log(Area)	1433.057	0.4881690	2.750345	0.2037514
92	TRUE	TRUE	log(AR)+log(Area)	1431.312	0.5413625	2.863410	0.2015637
93	TRUE	TRUE	log(AR)+log(Area)	1430.271	0.5385916	2.848772	0.2022597
94	TRUE	TRUE	log(AR)+log(Area)	1431.421	0.5113514	2.791561	0.2024096
95	TRUE	TRUE	log(AR)+log(Area)	1430.158	0.5295532	2.823935	0.2041853
96	TRUE	TRUE	log(AR)+log(Area)	1431.366	0.5194336	2.809109	0.2060413
97	TRUE	TRUE	log(AR)+log(Area)	1431.624	0.5271306	2.831022	0.2046725
98	TRUE	TRUE	log(AR)+log(Area)	1433.633	0.4841528	2.745334	0.2024445
99	TRUE	TRUE	log(AR)+log(Area)	1434.379	0.4618785	2.699150	0.2031756
100	TRUE	TRUE	log(AR)+log(Area)	1432.368	0.5146963	2.807883	0.2004202
